# Modulating the Interactions of Peptide‐Polyphenol for Supramolecular Assembly Coatings with Controllable Kinetics and Multifunctionalities

**DOI:** 10.1002/advs.202412194

**Published:** 2024-11-25

**Authors:** Kaiyuan Huo, Wenjie Liu, Zeyu Shou, Hongxiang Wang, Hao Liu, Yang Chen, Xingjie Zan, Qing Wang, Na Li

**Affiliations:** ^1^ School of Ophthalmology and Optometry School of Biomedical Engineering Wenzhou Medical University Wenzhou Zhejiang 325027 China; ^2^ Wenzhou Institute University of Chinese Academy of Sciences Wenzhou Key Laboratory of Perioperative Medicine Wenzhou Zhejiang 325001 China; ^3^ School of Materials Science and Engineering Zhengzhou University Zhengzhou 450001 China; ^4^ Department of Orthopedics The People's Hospital of Zhuji Affiliated Zhuji Hospital Wenzhou Medical University Shaoxing Zhejiang 311800 China; ^5^ Department of Orthopedics The First Affiliated Hospital of Wenzhou Medical University Wenzhou Zhejiang 325000 China; ^6^ School of Pharmacy Zhejiang Chinese Medical University Hangzhou Zhejiang 310053 China; ^7^ Yongkang First People's Hospital of Wenzhou Medical University Yongkang Zhejiang 321300 China

**Keywords:** coatings, multifunctionalities, peptides, polyphenols, supramolecular assembly

## Abstract

Polyphenols and peptides represent two fundamental building blocks in the kingdom of supramolecular assembly (SA) coatings, which have recently attracted considerable interest. Regulating the assembly kinetics of SA coatings is critical to controlling the performance of SA coatings, but this area is still in its infancy, especially in the SA coating of peptide‐polyphenol. Herein, a library of oligopeptides with rich diversity, numerous polyphenols, and modulators are explored to reveal their roles in the formation and regulation of SA coating. Citric acid (CA) is an effective regulator of interaction between the polyphenols and peptides to produce peptide‐polyphenol coatings, TCP. The electrostatic interaction between tannic acid (TA) and cationic peptide drives the formation of TCP, while the multiple hydrogen bonds between CA and TA and peptide dominate the assembly kinetics. With optimized assembly pH and the mass ratio of TA, CA, and peptide, the thickness of TCP coating deposits onto diverse substrates (glass, silica, titanium, polystyrene) is ≈400 nm with controllable kinetics. The multifunctional TCP coatings are endowed via peptide‐coupled functional units, including enhanced cellular adhesion, elevated osteogenic capacity, anti‐protein adsorption, and antimicrobial. This work contributes to the understanding of the assembly kinetics and functionalization of peptide‐polyphenol coatings.

## Graphical abstract

Citric acid is an effective modulator of polyphenol‐peptide interactions and can be used to prepare peptide‐polyphenol supramolecular self‐assembled coatings. The peptide‐coupled functional units confer multifunctionality to the coatings, including enhanced cell adhesion, improved osteogenesis, resistance to protein adsorption, and antimicrobial properties.

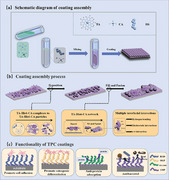

## 1 Introduction

Supramolecular assembly (SA) represents an intriguing mode of structural organization, based on the formation of complex architectures through non‐covalent interactions between simple structural units. This assembly strategy is widely observed in biological systems, where it plays a crucial role in constructing intricate functional structures.^[^ ^1^, ^2^ ^]^ Compared to traditional methods, coatings produced by SA have attracted widespread attention in recent years due to the avoidance of complex synthesis steps and the use of harmful cross–linking agents.^[^ ^3^, ^4^ ^]^ The diverse building blocks, the dynamic and flexible process, and the ability to integrate the physicochemical properties of building blocks confer a multitude of unique properties on SA coatings, including reversibility, adaptability, degradability, self‐healing, and stimulus responsiveness.^[^ ^5^, ^6^, ^7^ ^]^

Polyphenols exhibit considerable potential as fundamental building blocks for the fabrication of SA coatings due to their exceptional adhesive ability to various substrates, which was the key for SA coating, and a diverse range of biological activities, such as antibacterial, anti‐inflammatory, and antioxidant.^[^ ^8^, ^9^ ^]^ Importantly, polyphenols are capable of forming SA coatings through a variety of interactions, including hydrogen bonding, electrostatic interactions, and hydrophobic interactions with themselves or other functional molecules.^[^ ^10^, ^11^ ^]^ Synthetic peptides are another crucial building block in SA coatings, as their modular structure and high degree of design freedom permit a diverse range of applications^[^ ^12^ ^]^ in cellular signaling, immunomodulation, etc.^[^ ^13^ ^]^ The diversity of peptide side chains allows peptides to form a multitude of SA systems, including particles, micelles, and hydrogels, through non‐covalent interactions such as hydrogen bonding, electrostatic interactions, and π–π stacking, with themselves or other assemble units.^[^ ^14^, ^15^, ^16^ ^]^ The construction of peptide and polyphenol SA coatings is anticipated to achieve multifunctionalities based on the significant advantages of these two functional modules. For example, Han et al. constructed an SA network of polyphenols and polypeptides on a calcium carbonate template to collect polyphenol‐polypeptide vesicles with promoting cellular penetration and endosome escape properties. Zhao et al. engineered the peptide‐polyphenol coating by layer‐by‐layer technique, with antioxidants, promoted cell proliferation and differentiation, and enhanced in vitro osteogenesis and in vivo bone formation.^[^ ^17^ ^]^

The modulation of physicochemical properties, such as morphology, composition, stability, etc. is crucial for the performance of SA coatings, which can be manipulated through the assembly kinetic processes as well as the modulating of interactions between the building blocks.^[^ ^18^ ^]^ However, this research area is still in its nascent stage, with previous studies focusing on the SA coating of metal‐polyphenol‐networks (MPN). For example, Zhong et al. demonstrated a kinetic strategy for accelerating the growth of MPN films by modulating the oxidation process from TA‐Fe (II) to TA‐Fe (III) via reactive oxygen species.^[^ ^19^ ^]^ Rahim et al. showed etching iron (III) from rusty iron products was an effective method for continuous assembly MPN.^[^ ^20^ ^]^ Undoubtedly, a strategy for regulating the interactions between the constituent modules and the assembly kinetics will assist with the engineering of SA coatings.^[^ ^21^ ^]^ However, to the best of our knowledge, there have been no reports on modulating polyphenol‐peptide interactions to form SA coatings with controllable kinetics. Kinetic factors have been found to be important for synthesizing and controlling coordination‐driven structures and functions. For example, the assembly kinetics can strongly influence the mode of molecular packing, where different synthesis pathways can often lead to distinct self‐assembled structures with unique morphologies and functions.^[^ ^22^ ^]^ The rich diversity of polypeptides and the presence of multiple non‐covalent interactions between peptides and polyphenols (electrostatic interactions, hydrogen bonding interactions, etc.) introduces a degree of complexity to regulating between polyphenols and peptides.^[^ ^23^, ^24^ ^]^ Besides, the forces between peptides and polyphenols are highly variable, e.g., the forces between cationic peptides and polyphenols are close to those of covalent bonds,^[^ ^23^, ^24^ ^]^ which presents a significant challenge in identifying a suitable regulatory molecule.

**Scheme 1:**
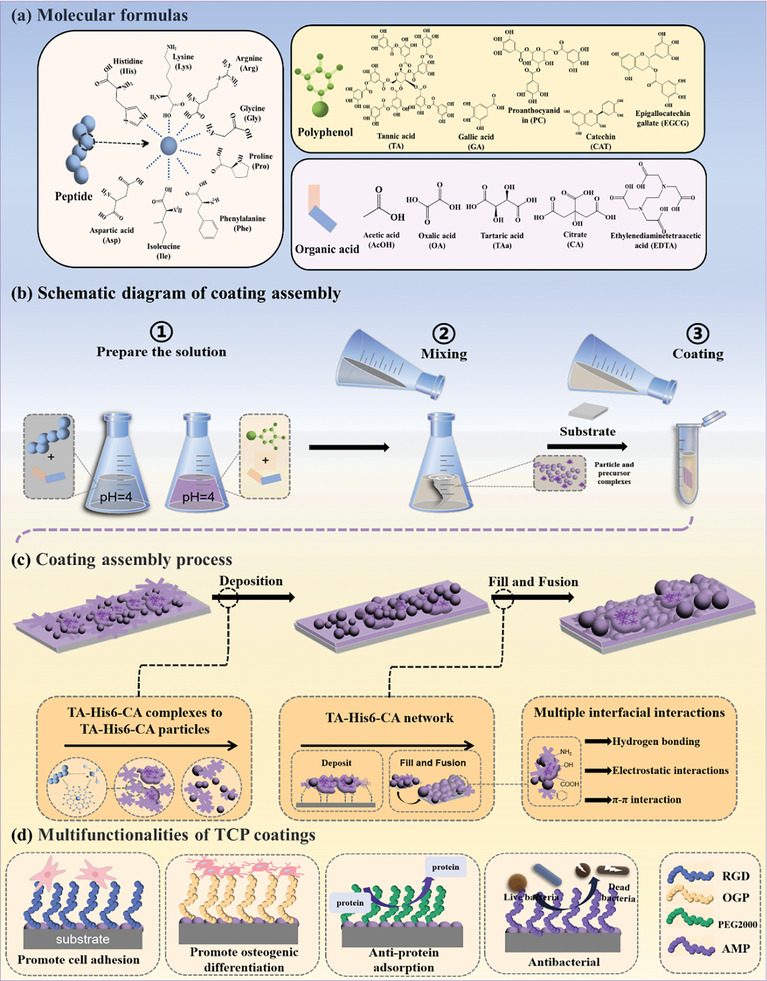
a) The molecular structural formula of peptide, polyphenol, and organic acid. b) Schematic diagram of the coating assembly process. c) Coating formation process. d) Schematic representation of the biological functions of TCP (TCH‐RGD/OGP/PEG/AMP) coatings.

Herein, a library of oligopeptides, including anionic peptides (Asp6), neutral hydrophilic peptides (Gly6), neutral hydrophobic peptides (Ile6 and Phe6), specialized structural peptides (Pro6), cationic peptides (Lys‐6, Arg‐6, and polyhistidine with different chain lengths (H2, H3, H6, and H‐9)) was designed to investigate the role of the rich diversity of peptides on the formation of SA coating of polyphenol‐peptide. Furthermore, various organic acids containing different numbers of carboxylic acid units, such as acetic acid (HAc), oxalic acid (OA), tartaric acid (TAa), citrate (CA), ethylenediamine tetraacetic acid (EDTA) were explored to find out the suitable regulator. And, a range of polyphenols (tannic acid TA, proanthocyanidins PC, epigallocatechin gallate EGCG, gallic acid GA, catechol CAT) were investigated to explore the generalizability of this strategy. Our data demonstrated that CA served as an effective modulator between TA and cationic peptides for fabricating TA‐CA peptide coatings (TCP) (**Scheme** **1**a). TCP coating, comprising TA, peptide, and CA, were successfully engineered by a one‐step process of mixing TA‐CA mixture with the CA‐peptide mixture on to diverse substrates (glass, silica, titanium, polystyrene) (Scheme 1b). Importantly, CA provides controllable kinetics for TCP formation, undergoing the deposition of TCP precursors followed by their integration into the TCP network (Scheme 1c), with the final assembly thickness reaching 400 nm. Depending on functional units linked with cationic peptides, the multifunctionalities could be imparted to the TCP coating, including promoting cellular adhesion, elevated osteogenic capacity, anti‐protein adhesion properties, and antimicrobial characteristics (Scheme 1d). This strategy offers a straightforward method for modulating the assembly kinetics and functionalizing the SA coating. The abbreviations of the relevant materials used in this work and the names of the prepared coatings are shown in Table S1 (Supporting Information).

## 2 Results and Discussion

### 2.1 Preparation and Characterization of Coatings

In order to find the modulator quickly, the ability to form SA coating on the substrate was used as the sole criterion by testing the change of thickness. All added materials had the same mass. Firstly, the role of the rich diversity of peptides on the formation of SA coating of polyphenol‐peptide was investigated. In the case without any modulator, no formation of SA coating was observed in the range of pH 3–11 (Figure S1, Supporting Information). Regulate the assembly process using CA, as shown in Scheme 1b, the assembly process of the SA coatings was carried out by mixing two solutions: Solution 1 (a mixture of TA and CA) and Solution 2 (a mixture of CA and peptide), where the silicon substrate was immersed for 24 h, at pH range 3–11. All cationic peptides were capable of forming SA coatings, and the pH applicable to the SA coatings formed varies for different cationic peptides (Figure S2, Supporting Information). Other peptides, including anionic peptides (Asp6), neutral hydrophilic peptides (Gly6), neutral hydrophobic peptides (Ile6 and Phe6), and specially structured peptides (Pro6), do not form coatings (Figure S2, Supporting Information). Obviously, the strong interaction between cationic peptide and TA was the precondition. When the regulator CA was replaced with other organic acids containing different numbers of carboxylic acid units (HAc, OA, TAa, EDTA), in the case of cationic peptide H6 and TA, no coating was formed (Figure S3, Supporting Information). The reason why CA can play a regulatory role will be revealed in subsequent studies. For the investigated polyphenols (TA, PC, EGCG, GA, CAT), in the case of cationic peptide H6 and CA, both TA and PC were formed in the coating (Figure S4, Supporting Information). As reported, the binding force between PC or TA and cationic peptide was much stronger than that of EGCG, GA, and CAT.^[^ ^25^ ^]^ This might be the reason for the failure of generating coating for EGCG, GA, and CAT. Gathering the above data, the system of TA, CA, and cationic oligopeptides were focused in the following investigation.

**Figure 1:**
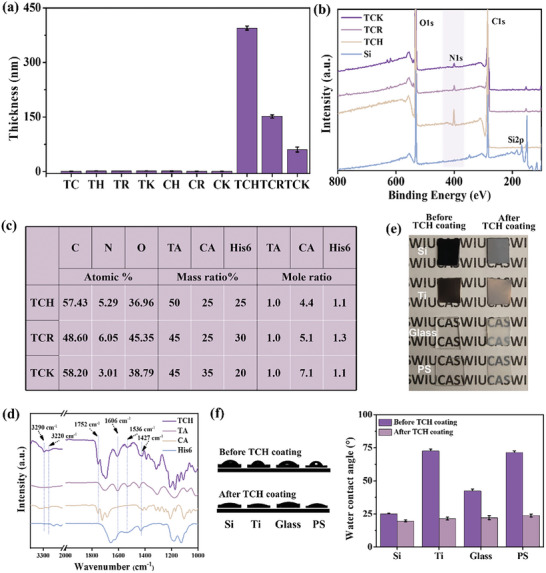
a) The thickness of the coatings after immersing silica into the solution of TCP (TCH, TCR, and TCK) and a two‐by‐two combination of TA, CA, and peptide solutions (pH 4) for 24 h. b) XPS spectra of bare Si substrate and TCH, TCR, and TCK coatings on Si. c) Atomic ratios of C, N, and O, the mass ratio as well as the molar ratio of TA, CA, and peptides in the three TCP (TCH, TCR, and TCK) coatings, measured by XPS. d) FTIR spectra of TCH, TA, CA, and H6. e) Optical images of Ti, Si, glass, and PS substrates before and after TCH coatings. f) Images (left) and values (right) of water contact angles of substrates (Ti, Si, glass, and PS) before and after TCH coatings.

As shown in **Figure** **1**a, after 24 h of immersion, all three cationic peptides (H6, Arg6, and Lys6) formed coatings on the silicon with thicknesses of 400 nm for TCH, 150 nm for TCR, and 60 nm for TCK, respectively. To determine that these three components are essential for assembly, we mixed the aforementioned components (polyphenols, organic acids, peptides) in pairwise combinations. During the same immersion time, almost no coating formation was observed on the substrate (Figure 1a). This indicates that each of these three components is essential for the sustained assembly of the coating. In XPS spectra, the presence of the N 1s signals in TCP (TCH, TCR, and TCK) coatings indicated the successful introduction of three peptides into their corresponding coatings. After coating, the disappearance of the characteristic signal (Si 2p) belonging to the substrate suggested that the coating was uniformly and densely assembled on the substrate (Figure 1b). Based on the composition of the C, N, and O elements of the three coatings in Figure 1c and combined with the molecular structural formulas of TA, H6, and CA, the percentages of these three components in the TCH coating were roughly calculated. As listed in Figure 1c, the composition of the three substances (TA, H6, and CA) varied in the three TCP coatings. In terms of mass, TA was the most abundant of all TCP coatings, followed by CA and peptides. With respect to molar ratio, however, CA exhibited the greatest abundance, with TA and peptides displaying similar amounts of substance. In TCH coating, the mass ratios of TA, H6, and CA were ≈2:1:1 (Figure 1c). The TCH coating was found to possess the highest thickness of the three TCP coatings, thus prompting subsequent studies to concentrate on this particular layer. The coating composition was further investigated by FTIR spectroscopy (Figure 1d). In the TCH coatings, the N─H stretching vibrations in the amide at 3290 and 3220, 1427 cm^−1^ correspond to the absorption bands of the amide III band (C─N), which provides evidence of the successful introduction of H6. The peaks centered at 1606 and 1532 cm^−1^ are attributed to the absorption of TA benzene. The 1752 cm^−1^ peak is attributed to the special C = O stretching vibration in the carboxyl group of the CA molecule, which proves the presence of CA in the coatings. The TCP could be coated onto a variety of substrates, including polymer (PS), inorganic (glass and silicon), and metal (Ti), as was evident from the optical images of the changes in transparency (Figure 1e) and the water contact angle (Figure 1f). The water contact angles of the silicon coated with TCH decreased from 24.98° to 19.78 °, the titanium coated with TCH decreased from 72.62 ° to 21.49 °, and the glass coating coated with TCH decreased from 42.34 ° to 22.24 °, and the plastic coated with TCH decreased from 71.51 ° to 23.79 °, indicating the TCH could be effectively coated onto various materials.

### 2.2 Factors on the Growth and Assembly Process of TCP Coating

**Figure 2:**
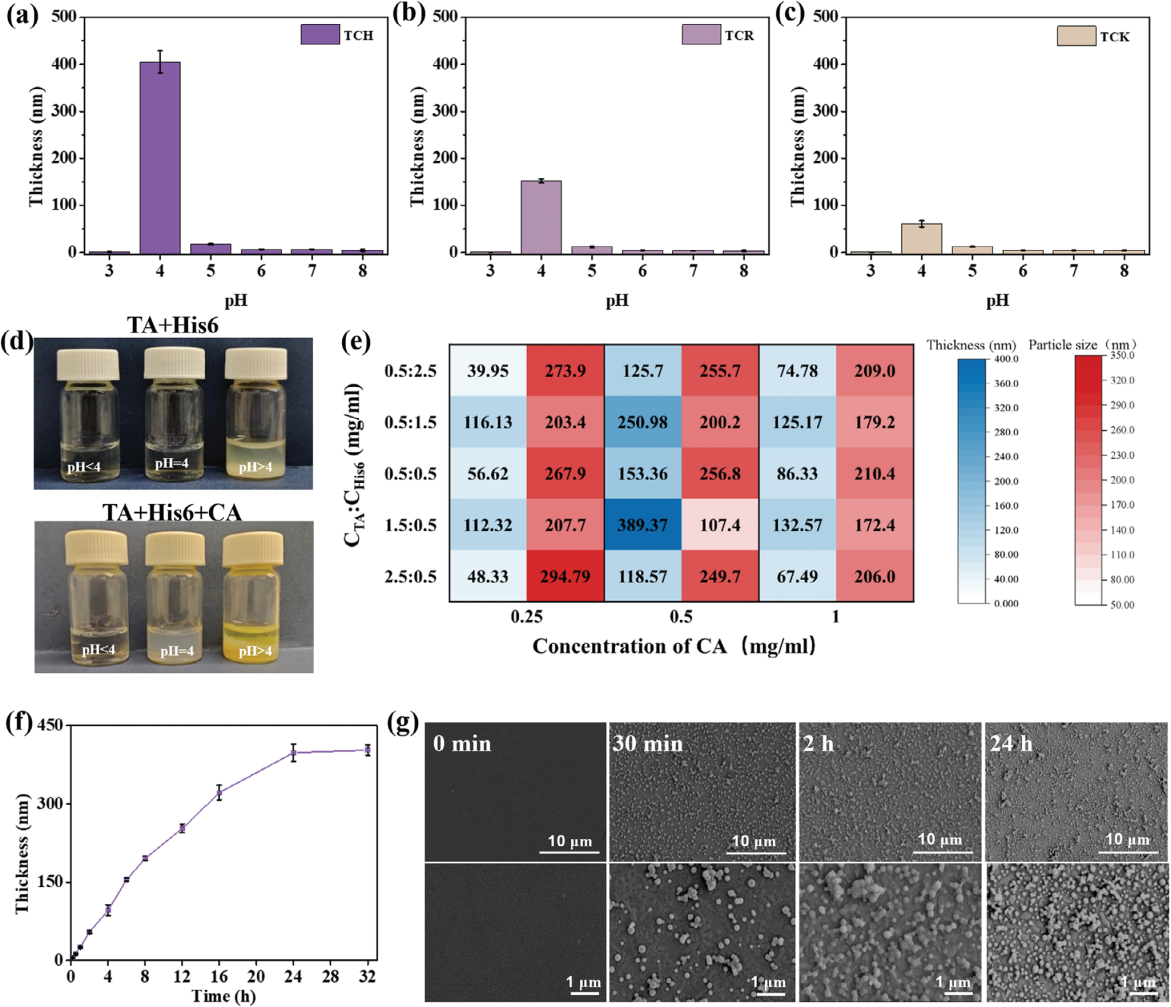
The coating thickness formed onto silica substrate after immersing into the solutions of a) TCH, b) TCR, and c) TCK, at different pH values for 24 h. d) Optical images of the mixture of TA and H6 at pH < 4, pH = 4, and pH > 4 in the absence and presence of CA. e) The effect of the composition of the three components (TA, CA, and H6) in solution on the TCH coating thickness (blue) formed onto silica at pH = 4 for 24 h and the initial size (red) of TCP complex in solution. The deeper color indicated the higher thickness or the larger particle size. f) The TCH coating thickness formed onto silica substrate versus the immersing time, at pH 4. g) SEM images of TCH coating formed onto silica at different assembly times. The scale bars in (g) were 10 µm (top panel) and 1 µm (bottom panel).

The thickness of TCH, TCR, and TCK coatings showed a strong dependence on pH, and the findings indicated that a pH 4 represented the optimal pH condition for the assembly of the three coatings, TCH, TCR, and TCK (**Figure** **2**a–c). Interestingly, the polyhistidine with different chain lengths (H2, H3, and H9), could be used to generate the TCP coatings (Figure S5, Supporting Information), but the optimal pH values increased with the increased chain length. To reveal the role of CA in the assembly process, the TCH was selected for further study. Without the presence of CA, the mixture of TA and H6 solution is completely clear at pH ≤ 4, but at pH > 4, a large amount of flocculation appeared in the solution rapidly (Figure 2d). In the presence of CA, the same phenomena were observed at pH < 4 and pH > 4 (Figure 2d). At a pH = 4, uniform nanoparticles are formed in the solution with an obvious Tyndall effect (Figure 2d and Figure S6, Supporting Information), due to the deprotonation of TA at pH 4.^[^ ^26^, ^27^, ^28^, ^29^ ^]^

Besides the pH, the composition of the three components (TA, CA, and H6) in the solution exhibited a significant effect on the growth of TCH coatings. The impact of the composition of the three components on the ultimate coating thickness was examined at a pH level of 4. Following a 24 h assembly period, the coatings were assembled at varying concentration ratios. However, the thickness of the coatings exhibited considerable variation (Figure 2e), with the thickest coatings reaching ≈400 nm (at a concentration of 0.5 mg mL^−1^ CA and a 3:1 ratio of TA to H6) and the thinnest coatings being ≈40 nm (at a concentration of 0.1 mg mL^−1^ CA and a 1:5 ratio of TA to H6). Although there is no clear pattern in the relationship between coating thickness and composition, it can be seen that for a fixed CA concentration, TCH coating thickness has a clear negative correlation with the initial particle size formed in the TCH solution (Figure 2e; Figure S7, Supporting Information). That is to say, the smaller the particle size in the assembly solution, the thicker the coating formed by the assembly. At pH 4 and a concentration of 0.5 mg mL^−1^ CA, the different ratios between TA and H6, the particle size of TCH, and the polydispersity index (PDI) of particles were monitored (Figure S8, Supporting Information). The size and PDI of the TCP remained relatively stable during the initial hours, followed by a pronounced increase after some hours (Figure S8, Supporting Information). This sharp change in size and PDI was observed across different solutions with varying proportional compositions, exhibiting no discernible pattern. The curve of TCP thickness versus time demonstrated a gradual increase during the initial stage, followed by a plateau (Figure S8, Supporting Information). The cessation point appeared to align with the point of the sharp increase in size and PDI (Figure S8, Supporting Information). These data suggested that the growth of coating thickness was closely related to the formation of TCP complexes in solution.

Under optimal assembly conditions (pH 4, a concentration of 0.5 mg mL^−1^ CA, and a 3:1 ratio of TA to H6), the thickness of TCP coated on silicon, grew gradually (Figure 2f), which could be further supported by the increased absorption in their UV–vis spectra (Figure S9, Supporting Information). The SEM was employed to monitor the growth of TCH by observing the morphology of TCH coated onto silicon at different immersing time scales. As shown in Figure 2g, prior to coating, the surface of the silicon substrate was flat. The morphology with deposited particles was observed at all tested times, and the surface became rougher and rougher with the increase in incubation time. In the magnified images at 0.5 h immersing, a few deposited particles appeared on the surface of the silicon substrate. Further deposition of particles and fusion of previously deposited particles were observed at 2 h. At 24 h, more particles accumulated onto the substrate, with more fused particles could be observed beneath the top aggregated particles. From the SEM images, during the process of generating TCP coating, the surface seemed rougher and rougher with the immersing time, which could be supported by the AFM test (Figure S10, Supporting Information). The roughness index (Ra) for the TCH coating assembled for 2 h was 17.6 nm, and increased to 39.1 nm of coating assembled for 24 h, further confirming the increased roughness with the coating growth, as observed in SEM (Figure 2g).

### 2.3 Growth Mechanism of TCH Coating

**Figure 3:**
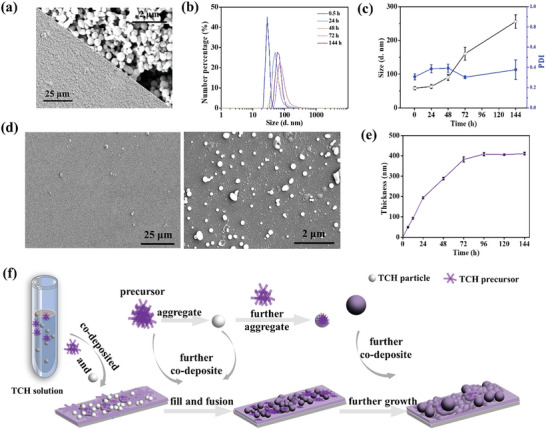
a) The SEM images of TCH particles separated from the initial solution of TCH by centrifuge; b) The size distribution curve, c) the size and PDI of the precursor at different standing times, tested by DLS. d) The TCH coating thickness formed onto the silica substrate versus the immersing time into the TCH precursor. e) SEM image of the coating formed from the precursor in the supernatant solution isolated from the initial solution of TCH. f) Schematic diagram of the growth mechanism of TCH coating. The scale bar in the lower left image of (a) is 25 µm, and in the upper right image, it is 2 µm. The scale bar was 25 µm in the left images and 2 µm in the right images for (d).

To better understand the assembly mechanism of TCH coating, inspired by the above observations, the TCH particles were separated from the initial solution of TCH by centrifuge. At the bottom of the centrifuge tube, TCH pellets were observed (Figure S10, Supporting Information). As observed by SEM (**Figure** **3**a), these particles were tightly bonded to each other, and no deformation was found in these particles, indicating that the TCP particles were relatively rigid and sticky with each other. A clear supernatant with a very strong Tyndall effect (Figure S11, Supporting Information) is observed, indicating that the supernatant contains many small particles, here called precursors. The size of the precursor in solution gradually increases over time from beginning 58 to 260 nm after standing for 144 h (Figure 3b,c), and PDI showed no obvious change in all the tested periods (Figure 3c). It is interesting to note that when a silicon wafer was placed in the precursor solution, it was still able to gradually grow a TCH coating with the same thickness (Figure 3d) as the TCH coating fabricated from the original solution (Figure 2f). However, it did not reach a plateau until 72 h (Figure 3d), which took longer than the original TCH solution (≈24 h in Figure 2f). In the SEM image of TCH surface morphology from the precursor solution (Figure 3e), it could be seen that the precursor‐formed coating has a similar morphology to the original coating (Figure 2g), but with fewer particles on the surface. However, the centrifuged particles could not continue to assemble into a coating after being redispersed (Figure S12, Supporting Information). It is clear that the TCH coating formation process was a precursor‐dominated process. Based on the above observations, the assembly mechanism of TCH coating was proposed and schematically illustrated in Figure 3f. When combining CA with H6 and TA, both the particles and precursors were generated in the solution. These particles were deposited along with the precursors. Meanwhile, the precursors subsequently aggregated to form new particles, and the precursors also adhered to the original particles, thereby increasing the size of the latter. The further deposition of particles with increased size led to the roughening of the coating surface. The coating stopped growing when the precursors were totally depleted.

### 2.4 The Interactions Inside TCH Coatings

**Figure 4:**
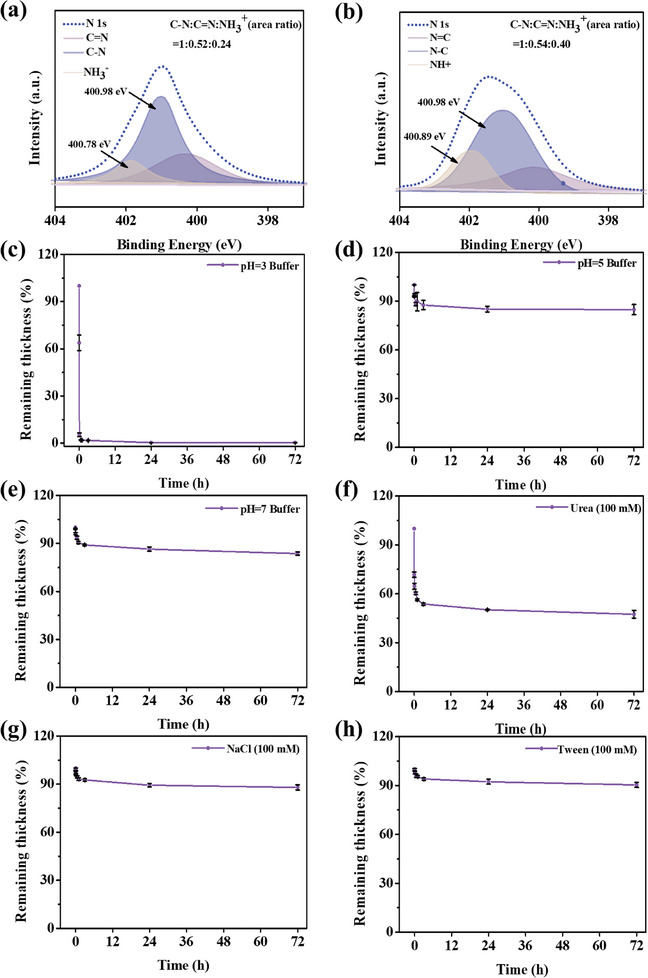
Split peak of N1s in a) H6 and b) TCH coatings. The relative thickness of the TCH curve over time was observed in a series of solutions including c) pH 3 buffer solution, d) pH 5 buffer solution, e) pH 7 buffer solution, f) 100 mm urea solution, g) 100 mm NaCl solution and h) 100 mm Tween 20 solution.

The XPS data of the raw materials and TCH coatings were analyzed to investigate the intermolecular interactions between the coatings. In the split peak of N1s, the binding energy of the ─NH_3_^+^ in H6 is 400.78 eV (**Figure** **4**a),^[^ ^30^ ^]^ and the area percentage of ─NH_3_^+^ to total C─N was ≈24%. In the TCH coatings (Figure 4b), the binding energy of the ─NH_3_^+^ increased to 400.89 eV, and the area percentage increased to 40%. These data provide compelling evidence for the presence of electrostatic interactions in TCH. In addition, the area percentage of C═N didn't show obvious variation in H6 and TCH coating, suggesting no newly formed Schiff base bonds (C═N) in TCH coating. In order to gain deeper insight into the intermolecular forces present in the coatings, a variety of solutions were employed to subject the samples. These included buffer solutions with varying pH values, NaCl solutions, urea solutions, and tween 20 solutions, which were used to study the coatings' electrostatic interactions, hydrogen bonding, and hydrophobic properties, respectively. At a pH value of 3, the TCH coating degrades rapidly (Figure 4c). In contrast, at pH values of 5 and 7 (Figure 4d,e), the coatings demonstrated relative stability. The strongly pH‐dependent behavior suggested there was an electrostatic interactions in TCH. The complete decomposition of TCH coating at pH 3 was attributed to the protonation of TA, which greatly reduced the electrostatic interaction between H6 and TA. In the urea solution (Figure 4f), the decomposition of the TCH coating was also observed, but there was still a residue of 58% after 72 h of immersion. In a 100 mm NaCl solution or a 100 mm Tween 20 solution, the coating undergoes slight degradation. After soaking for 72 h, the coating loses ≈12% in 100 mm NaCl and ≈10% in 100 mm Tween 20 (Figure 4g,h). The data of stability in different solutions suggested the intermolecular interactions in the coating were dominated by electrostatic forces, with hydrogen bonding interactions and weak hydrophobic interactions. It is noteworthy that due to the presence of acidic microenvironments in certain pathological tissues (including tumor tissues) and cellular lysosomes, the phenomenon of rapid degradation of the coating at low pH values may be utilized in the field of drug delivery.^[^ ^31^, ^32^ ^]^

### 2.5 The Role of CA in TCH Coating

**Figure 5:**
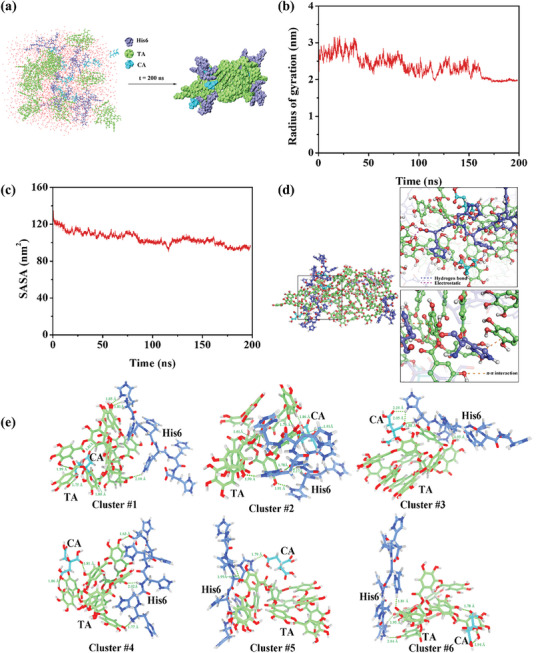
Under the MD simulation, a) Schematic representation of the formation of nano‐aggregates after 200 ns assembly of TA, CA, and H6 (a total of 6 molecules randomly populated into an 8 × 8 × 8 nm3 box) from the initial stage. b) the gyration radius and c) SASA of the formed nanocluster with the simulated time; d) The stabilization of TA‐CA‐H6 nano‐assemblies is facilitated by a range of non‐covalent interactions, including electrostatic interactions, hydrogen bonding, and π–π stacking (illustrated by blue, purple, and orange dashed lines, respectively). e) Intermolecular interaction patterns and conformations of the first six lowest energy molecular clusters in the H6‐TA‐CA system derived from quantum chemical calculations.

In the MD simulation, the TA, CA, and H6 could self‐assemble to form stable nano‐clusters (**Figure** **5**a), in which TA self‐aggregated in the middle due to its intermolecular interactions (hydrogen bonding and hydrophobic interactions), and H6 surrounded by CA stably bound to the TA aggregates. The compactness of TA‐CA‐H6 nano‐clusters could be seen from the density distribution along the nano‐clusters (Figure S13a, Supporting Information). The density of the nanocluster was higher near the center, which is ≈200 kg m^−3^, while it is significantly lower away from the center of the box. The radius of gyration and solvent‐accessible surface area (SASA) of the nano‐cluster can be used to evaluate the progress and tightness of the system assembly. As shown in Figure 5b,c, as the generation of the nano‐clusters, the values of gyration and SASA gradually decreased. The relatively high gyration and SASA values in the beginning stage indicated that the molecules in the system had a large relative motion in this period of time, and the molecules are constantly searching for the appropriate binding conformation. After 180 ns, both the gyration and SASA values reached a plateau, the gyration and SASA were ≈1.699 ± 0.021 nm and 90 nm^2^, respectively. Compared to the original, both gyration and SASA were decreased to ≈35%, indicating a stable assembly was formed. The intermolecular interactions within the nano‐clusters were analyzed, and their representative interactive modes were illustrated in Figure 5d. This analysis revealed the presence of electrostatic and π–π interactions between H6 and TA, in addition to hydrogen‐bonding interactions between CA and TA as well as CA and H6. To facilitate the calculation, a single molecule each of TA, CA, and H6 was employed in order to calculate the binding energies. The binding energy of TA with H6 was ≈−2047.32 ± 145.74 kJ mol^−1^. The binding energies of CA with H6 and TA were found to be −218.99 ± 92.49 and −450.64 ± 108.25 kJ mol−^1^, respectively (Figure S13b, Supporting Information). Under optimal assembly conditions (TA:CA: H6 molar ratio of 1:4.4:1.1), CA is present in large quantities. It might thus be inferred that the number of hydrogen bonds formed between CA and TA and H6 was considerably greater than that of the electrostatic bonds formed between TA and H6, which represented a significant factor in the success of CA in regulating the electrostatic interaction between TA and H6. To further investigate the regulatory role of CA during assembly, the binding mode and the corresponding interaction energy between TA, H6, and CA were investigated using quantum chemistry. The results of the structural optimization and vibrational frequency calculations of TA, H6, and CA molecules were presented in Figure 5e, which displayed the six conformations with the lowest energies selected from 100 conformations. The results demonstrated that CA engages in hydrogen bonding interactions with both TA and H6 and that CA regulates the interaction between TA and H6 through the formation of hydrogen bonds. Both MD simulations and quantum chemical calculations illustrated the existence of a large number of hydrogen bonding interactions between CA and H6 and TA, which modulated the electrostatic force between TA and H6. These findings were consistent with the above experimental data.

### 2.6 Engineering TCH Coating With Multi‐Functionalities

The interfacial characteristics of biomedical materials are intimately linked to a multitude of biological phenomena occurring under physiological conditions. These include early protein adsorption, microbial infection, cell adhesion, cell proliferation rate, integration of the implant into the surrounding tissue, and the development of an immune response in the mid‐ and late stages. The occurrence of any of these events can have a profound impact on the biomedical outcome and fate of the medical material. The specific requirements for biomedical materials with respect to their interfaces depend on the intended application of the material in question. For instance, in the context of bone implants, the creation of a proliferative and osteogenic interface is vital to enhance the incorporation of the implant into the surrounding bone tissue, thereby optimizing the success of the implantation procedure. In the case of materials that come into direct contact with blood, interfaces with anti‐protein adhesion properties, anti‐coagulation characteristics, and efficient sterilization capabilities are essential. Consequently, it is of paramount importance to devise a straightforward and efficacious method for the customization of biomedical material interfaces with specific biological functions. The subsequent sections demonstrate that this coating methodology can be employed to generate a range of coating types with diverse functions by linking H6 with functional components. The functional units include the short peptide RGD, which facilitates cell attachment and proliferation; the peptide pOGP, which promotes osteogenic bone growth; the long peptide AMP, which exhibits antimicrobial properties; and the PEG2k, which exhibits anti‐protein adhesion properties (Figure S14, Supporting Information).

#### 2.6.1 Promote Cell Attachment and Proliferation by TCH‐RGD

**Figure 6:**
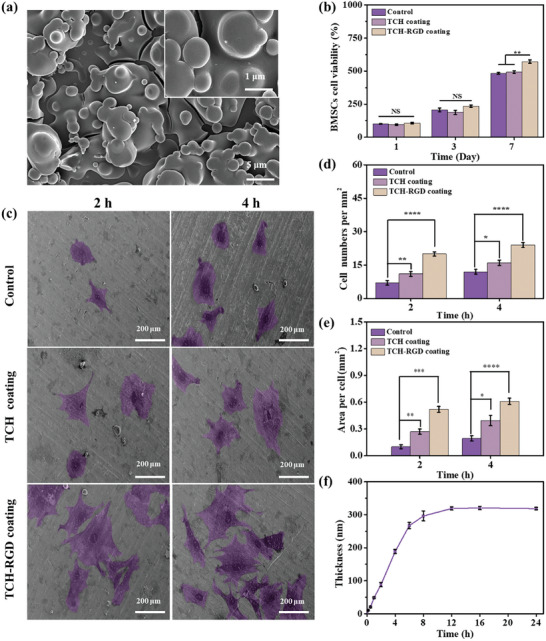
a) SEM microscope image of TCH‐RGD coatings, inset is a magnified image. b) Thickness of TCH‐RGD coatings versus incubation time, TCH‐RGD coatings were made by immersing silica into TA (1.5 mg mL^−1^), CA (0.5 mg mL^−1^), and H6‐RGD (0.5 mg mL^−1^) solutions with pH 4. c) SEM images of MC3T3‐E1 cells cultured for 2 and 4 h on titanium substrate, TCP coating and TCH‐RGD coating (The cell pseudo‐color in (c) was manipulated using Photoshop (PS) to better observe cell adhesion and spreading). d) Number of cells and e) area (per square millimeter) of MC3T3‐E1 cells attached to titanium substrate, TCP coating, and TCH‐RGD coating after 2 and 4 h. Data in (d) and (e) are averages of at least 50 MC3T3‐E1 cells. f) Cell viability of MC3T3‐E1 cells inoculated onto coverslips, TCH coatings, and TCH‐RGD coatings on days 1, 3, and 7. In Figure (a), The scale bare was 5 µm in the image and 1 µm for the magnified image. The scale bar was 200 µm for Figure (c). No significance was noted as “NS,” *p < 0.05, **p < 0.01, ***p < 0.001 compared with the Control group, using a *t*‐test.

RGD (Arg‐Gly‐Asp), a short peptide sequence that is widely found in extracellular matrix proteins, is able to bind to the integrin receptor on the surface of the cell and play a variety of biological roles, which is important for the promotion of cell adhesion, migration, proliferation, and differentiation.^[^ ^33^ ^]^ The application of RGD sequences to biological coatings has been extensively explored in recent years.^[^ ^34^, ^35^ ^]^ Both the SEM images of TCH‐RGD coatings (**Figure** **6**a) and growth curves of TCH‐RGD coating (Figure 6b) supported that the TCH‐RGD preparation was successful. The rapid attachment and spreading of early cells on the implant facilitate the integration of tissue cells, reduce the influence of bacteria, and are conducive to the successful implantation of bone implants.^[^ ^36^ ^]^ The TCH and TCH‐RGD coatings were applied to the Ti substrate, and the morphology of attached MC3T3‐E1 cells onto these samples was observed using SEM. The images obtained at 2 and 4 h post‐inoculation are presented in Figure 6c. Even after 4 h, the cells on the surface of the Ti substrate (control) did not exhibit complete spreading. In contrast, cells on the TCH and TCH‐RGD‐coated surface exhibited obvious spreading after 2 and 4 h. Furthermore, the TCH‐RGD coating exhibited clear evidence of cell peripheral pseudopods and new adhesion sites with the substrate. The statistical analysis of the number of attached cells per mm^2^ revealed a significant increase in the number of cells on the surfaces of the TCH and TCH‐RGD coatings in comparison to the number observed on the bare Ti surface (Figure 6d). The statistical analysis of the mean cellular area revealed that the TCH and TCH‐RGD coatings exhibited markedly larger areas than the bare Ti surface (Figure 6e). These findings indicated that the TCH‐RGD coating facilitated cell adhesion and suggested that the cells may have demonstrated enhanced survival characteristics. The viability of MC3T3‐E1 cells at 1, 3, and 7 days was examined using the CCK‐8 kit in order to assess the proliferative behavior of MC3T3‐E1 cells on TCH and TCH‐RGD coatings. In all samples, the viability of MC3T3‐E1 cells increased with the incubation time, indicating that all inoculated cells had settled and initiated the proliferation process (Figure 6f). The TCH‐RGD coating exhibited the highest number of cells among all the samples tested. The live/dead cell staining of MC3T3‐E1 cells cultured on glass coverslips (control), TCH, and TCH‐RGD coatings for 2 days, revealed no red color (indicative of dead cells) in any of the samples (Figure S15a,b, Supporting Information). The number of cells on TCH‐RGD coating was highest, followed by TCH coating and the glass coverslip, further supporting the above observation of proliferation assay (Figure 6f) These data were consistent with one another, indicating that the RGD was integrated into the coating and thereby promoting cellular attachment and proliferation.

#### 2.6.2 Promotion In Vitro Osteogenesis

**Figure 7:**
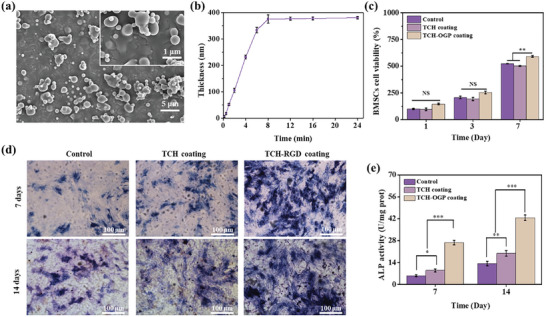
a) SEM images of TCH‐OGP coatings. b) The dependence of thickness on TCH‐OGP coating on the incubation time, the TCH‐OGP coating was fabricated by immersing silica into the solution of TA (1.5mg mL^−1^), CA (0.5mg mL^−1^) and H6‐OGP (0.5mg mL^−1^), at pH 4. c) Viability of MC3T3‐E1 cells inoculated on glass TCH and TCH‐RGD coatings at days 1, 3 and 7. d) Ti, TCH coatings and TCH‐OGP coatings induced osteogenic alkaline phosphatase staining at 7 and 14 days. e) Quantitative determination of ALP activity. In Figure (a), The scale bare was 5 µm in the image and 1 µm for the magnified image. The scale bar was 100 µm for Figure (d). The scale bar was 100 µm for Figure (d). No significance noted as “NS,” *p < 0.05, **p < 0.01, ***p < 0.001 compared with the control group, using a *t*‐test.

The successful construction of TCH‐OGP coating was verified by SEM (**Figure** **7**a) and the growth curve of the TCH‐OGP coating (Figure 7b). The MC3T3‐E1 cells were cultured on glass, TCH, and TCH‐OGP coatings. The viability of the MC3T3‐E1 cells was observed to increase with culture time in all samples, indicating that all inoculated cells had settled and commenced proliferation in a normal manner (Figure 7c). Moreover, the results of the live‐dead cell staining on day 2 (Figure S16a,b, Supporting Information) corroborate the above observation (Figure S16, Supporting Information). An in vitro osteogenic differentiation assay was conducted, during which ALP staining was performed as a marker of early osteogenesis. The osteogenic induction medium was introduced at the point when the cells reached 80% confluence. Subsequently, the ALP activity of MC3T3‐E1 cells was assessed following an additional 7 and 14 days of culture. In comparison to the control group, which exhibited only a minimal degree of light blue, the surface of TCH coatings and TCH‐OGP coatings displayed a markedly darker blue, indicating a high level of ALP expression in TCH coatings and TCH‐OGP coatings. The data clearly demonstrate that the ALP activity of the TCH‐OGP coating was significantly higher than that of the TCH coating. This provided strong evidence that the TCH‐OGP coating was more efficacious in promoting early osteogenesis of MC3T3‐E1 cells (Figure 7d). The quantitative analysis of ALP (Figure 7e) revealed significant differences between the control and TCH coatings and TCH‐OGP coatings, which further supported the above observation in Figure 7d. The superior osteogenesis observed in TCH‐OGP coatings may be attributed to the synergistic effect of CA and OGP on enhancing bone formation and promoting osseointegration.^[^ ^37^ ^]^

#### 2.6.3 Anti‐protein Adsorption

**Figure 8:**
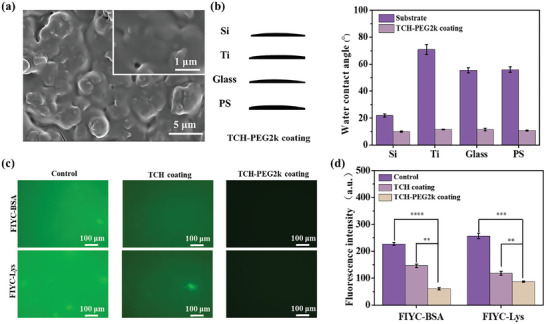
a) SEM image of TCH‐PEG20k coating. b) Water contact angle of TCH, TCH‐PEG2k coatings assembled on different substrates. c) Fluorescence images of the glass slide, TCH, TCH‐PEG2k after 48 h of immersion in fluorescently labeled bovine serum protein/lysin. d) Fluorescence intensity statistics. In Figure (a), The scale bare was 5 µm in the image and 1 µm for the magnified image. The scale bar was 100 µm for Figure (c). No significance noted as “NS,” *p < 0.05, **p < 0.01, ***p < 0.001 compared with the Control group, using a *t*‐test.

SEM (**Figure** **8**a) and growth curves of TCH‐PEG coatings (Figure S17a, Supporting Information) confirmed the successful construction of TCH‐OGP coatings. The WCA is an important index for evaluating the anti‐protein adsorption properties of a coating. It is generally accepted that a low WCA is favorable for anti‐protein adsorption.^[^ ^38^ ^]^ When TCH and TCH‐PEG2k coatings were applied to different substrates, the WCA of the TCH coating decreased to ≈18°, and the WCA of the TCH‐PEG2k coating exhibited a further decrease to ≈10° (Figure 8b; Figure S17b, Supporting Information). BSA and Lys were selected as model proteins for anti‐protein adsorption studies. BSA (isoelectric point 4.7–5.2, molecular weight 66.4 kDa) is representative of acidic proteins, while Lys (isoelectric point 11.0–11.5, molecular weight 14.3 kDa) is representative of basic proteins. After immersing glass, TCH, and TCH‐PEG2k coatings into FITC‐BSA and FITC‐Lys (at a concentration of 1 mg mL^−1^) for 48 h, Figure 8c illustrated a pronounced decline in the adsorption of BSA and Lys on TCH coatings relative to the control (glass slide). The adsorption of FITC‐BSA and FITC‐Lys was observed in TCH coatings, though this was barely discernible in TCH‐PEG2k coatings. A statistical analysis of the fluorescence intensity of the adsorbed proteins (Figure 8d) revealed significant differences between the control and TCH groups, as well as between the TCH and TCH‐PEG2k groups. These data suggested the PEG was successfully displayed on the surface and formed a hydrated and neutral PEG layer that prevented protein adhesion. The promising anti‐protein adsorption ability of TCH‐PEG2k indicated that the density of PEG in TCH‐PEG2k was sufficient to cover all areas of the substrates without notable defects.^[^ ^39^ ^]^

#### 2.6.4 Antimicrobial Properties

**Figure 9:**
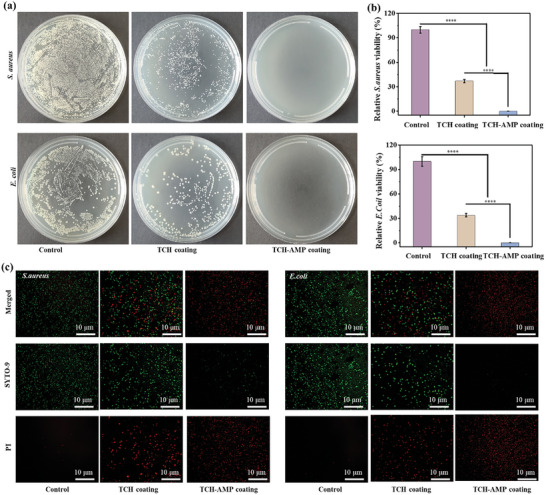
a) Photographs of typical agar plates of *S. aureus* and *E. coli* treated on glass coverslips, TCH‐coated and TCH‐AMP‐coated surfaces, and b) relative bacterial viability. c) Fluorescence images of live/dead staining using green fluorescence (SYTO9) and red fluorescence (propidium iodide, PI) for *S. aureus* and *E. coli* after 8 h of surface treatment with blanks, TCH‐coated, and TCH‐AMP‐coated surfaces are presented herewith. The scale bar was 10 µm for Figure (c). No significance noted as “NS,” *p < 0.05, **p < 0.01, ***p < 0.001 compared with the Control group, using a *t*‐test.

It is imperative that implant coatings exhibit antimicrobial resistance, as bacterial infection can lead to the failure of the implant, which can have disastrous consequences for the patient. The formation of TCH‐AMP can be confirmed by scanning electron microscopy (SEM) and coating growth curves (Figure S18a,b, Supporting Information). The antimicrobial property was investigated by incubating the coating with two types of bacteria: *S. aureus* (gram‐positive) and *E. coli* (gram‐negative). Regardless of the type of bacteria, the TCH coating demonstrated some bactericidal performance, but a small number of bacteria survived on the coating. In contrast, the survival of bacteria on the TCH‐AMP coating was almost invisible, indicating that the TCH‐AMP coating has the strongest bactericidal ability (**Figure** **9**a). The related statistical analyses revealed significant differences between the control and TCH coatings, as well as between the TCH coating and the TCH‐AMP coating (Figure 9b). To gain further insights into the bactericidal capacity of the TCH‐AMP coating, the bacteria were subjected to a live/dead staining assay, in which the live bacteria were stained with the green fluorescent nucleic acid stain SYTO‐9, while dead bacteria were stained with the red fluorescent nucleic acid stain PI. The *S. aureus* and *E. coli* were incubated with glass (the control), TCH, and TCH‐AMP coatings for 8 h, after which bacteria were rinsed three times with PBS, then stained and observed under an inverted fluorescence microscope. As illustrated in Figure 9c, almost no dead bacteria were observed for both *S. aureus* and *E. coli* on the glass sheet, indicating an absence of bactericidal activity. In contrast, the TCH‐AMP exhibited the complete absence of live bacteria, indicative of exceptional bactericidal efficacy. The TCH coating exhibited a mixture of live and dead bacteria, indicating a degree of bactericidal capacity. The results obtained with SYTO‐9 were in accordance with the data presented in Figure 9a,b, indicating the AMP was present on the surface and exhibited a strong antibacterial ability.

## 3 Conclusion

In conclusion, numerous peptides with rich diversity, a library of polyphenols, and different modulators were investigated to reveal their roles in the formation and regulation of SA coating of polyphenol‐peptide. Cationic peptides are preferred over anionic and neutral peptides for the formation of peptide‐polyphenol coatings. CA was an effective regulator for cationic peptide‐polyphenol interactions compared to other organic acids. The binding force between polyphenols and cationic peptides was directly related to the formation of polypeptide‐polyphenol coatings. TCP coating composed of TA, CA, and peptide, was successfully engineered onto a variety of substrates (glass, silica, titanium, polystyrene) based on the pH and the mass ratio of TA, CA, and peptide. A variety of cationic peptides (including H2, H3, H6, H9, R6, and K6) and cationic peptides linked with different functional units (H6‐OGP, H6‐PEG2k, H6‐AMP, and H6‐RGD) can be incorporated into the TCP coating. The TCP coatings underwent a deposition of TCP particles, which were subsequently filled into the precursor and fused to generate a TCP network. The generation of long‐term stable TCP precursors was identified as a critical factor in controlling the formation of TCH coatings. A multitude of intermolecular interactions were observed between TA, CA, and peptide. The electrostatic interaction between TA and cationic peptides was the main driving force for the TCP formation. CA proved effective in regulating the electrostatic interaction between TA and cationic peptides through multiple hydrogen bonds between CA and TA and peptide, which dominated the behaviors of the precursor and assembly kinetics and process. The linked functional units to H6 could be efficiently presented onto the CTP coating, which endowed the coatings with multifunctionalities, including enhanced cell adhesion, elevated osteogenic capacity, promising anti‐protein adsorption, and excellent antibacterial.

## 4 Experimental Section

### Chemicals and Materials

Tannic acid (TA, 99%), proanthocyanidins (PC,99%), epigallocatechin gallate (EGCG, 99%), gallic acid (GA, 99%), catechins (CAT, 99%), citric acid monohydrate (CA, 99%), acetate acid (HAc, 99%), oxalic acid (OA, 98%), tartaric acid (TAa, 98%), and ethylenediaminetetraacetic acid (EDTA,98%), acetic acid (AA, 98%), sulfuric acid (H2SO4, 98%), hydrogen peroxide (H2O2, 30%), tris(hydroxymethyl)‐aminomethane (Tris, 99%), hydrogen chloride (HCl, 38%), monosodium phosphate (NaH2PO4,99%), sodium phosphate dibasic (Na2HPO4, 99%), sodium chloride (NaCl, 99.5%), Urea (99%), Tween20 (>40%), sodium hydroxide (NaOH, >98%), ammonia (25%), and 4,6‐diamidino‐2‐phenylindole (DAPI) were purchased from Sigma. Paraformaldehyde (4%), Triton X‐100, sodium β‐glycerophosphate, and fetal bovine serum (FBS) were purchased from Solarbio Life Science. Vitamin C, Cell Counting Kit‐8 (CCK‐8). Ti, polystyrene (PS) was cleaned with anhydrous ethanol by ultrasonic cleaning (this process was repeated 3 times). Silicon wafers and 14 mm diameter round glass coverslips were washed with piranha solution (30% hydrogen peroxide and 70% concentrated sulfuric acid, V/V) for 2 h at 98 °C, followed by washing with anhydrous ethanol and deionized water under sonication and dried under nitrogen airflow. Fluorescently labeled bovine serum proteins (FITC‐BSA) and lysozyme (FITC‐Lys) were synthesized using a nucleophilic reaction by coupling FITC to the free ε‐amino group of BSA and Lys to form a thiourea bond, as reported.^[^ ^40^ ^]^ Peptide, including anionic peptide aspartate‐6 (Asp6), the neutral hydrophilic peptide glycine‐6 (Gly6), the neutral hydrophobic peptides isoleucine‐6 (Ile6) and phenylalanine‐6 (Phe6), the specialized structural peptide proline‐6 (Pro6), cationic peptides include lysine‐6 (Lys6), arginine‐6 (Arg6), and polyhistidine of different chain lengths: histidine‐2 (H2), histidine‐3 (H3), histidine‐6 (H6), and histidine‐9 (H9), and peptides linked with different functional units: H6‐OGP (H6‐GGYGFGGYGFGG), H6‐PEG2k (H6Cys‐mal‐PEG2k), H6‐antimicrobial peptide (AMP) (H6‐acp‐KWKLFKKIGAVLKVL‐NH2) and H6‐RGD (H6‐Acp‐RGD‐Acp‐RGD‐Acp‐RGD) were synthesized by Hangzhou Specialized Peptide Biology Co. Ltd (Hangzhou, China) with purity (> 95%) and used without further purification. The water used here was ultrapure water, with a resistivity of 18.2 MΩ cm.

### Preparation of TCP Coatings

Prepare two solutions: Solution 1: a mixture of polyphenols and organic acids. Solution 2: a mixture of peptides and organic acids. Concentration: Solution 1 was a mixed aqueous solution of 3 mg mL^−1^ polyphenols and 1.5 mg mL^−1^ organic acids, and Solution 2 was a mixed aqueous solution of 3 mg mL^−1^ peptides and 1.5 mg mL^−1^ organic acids. (Polyphenols include five types: TA, PC, EGCG, GA, and CAT. When preparing a polyphenol solution, choose one of these polyphenols for preparation.) (Organic acids include five types: HAc, OA, TAa, CA, and EDTA. When preparing a solution, choose one of these organic acids for preparation.) (Peptides include the following: His2, His3, His6, His9, Arg6, Lys6, Pro6, Ile6, Phe6, Gly6, Asp6. When preparing a solution, choose one of these peptides for preparation). Both solutions were adjusted to pH = 4–11 with 0.1 M NaOH or 0.1 M HCl. 0.4 mL of Solution 1 and 0.4 mL of Solution 2 were transferred to a 1.5 mL centrifuge tube and vortexed and mixed for 10 s. The substrate (silicon, titanium, glass, polystyrene) was immersed vertically in the mixed solution. After the preset time had elapsed, the substrates were removed, washed thoroughly with water, and blown dry with an air stream for subsequent testing. Depending on the peptide applied to the assembled coating, the coating of TA‐CA‐H2 was named TCH2, the coating of TA‐CA‐H3 was named TCH3, the coating of TA‐CA‐H9 was named TCH9, the coating of TA‐CA‐H6 was named TCH, TCR denotes TA‐CA‐Arg6, TCK denotes TA‐CA‐ Lys6, TCH‐ RGD denotes TA‐CA‐H6‐RGD, TCH‐OGP denotes TA‐CA‐H6‐RGD, TCH‐PEG2k denotes TA‐CA‐H6‐PEG2000, and TCH‐AMP denotes TA‐CA‐H6‐AMP.

### Factors on the Assembly of TCH Coatings

For the pH effect on the assembly of TCH coatings, the pH values of Solutions 1 and 2 were adjusted to 3, 4, 5, 6, 7, and 8. Following the same protocol of TCP coatings, the thickness, particle size, and polydispersity index (PDI) were recorded at preset time. For the exploration of the mass ratio effect of TA, CA, and H6 on the assembly of TCH coatings, the mass ratio of TA (1 mg mL^−1^) and H6 (5 mg mL^−1^); TA (1 mg mL^−1^) and H6 (3 mg mL^−1^); TA (1 mg mL^−1^) and H6 (1 mg mL^−1^); TA (3 mg mL^−1^) and H6 (1 mg mL^−1^); TA (5 mg mL^−1^) and H6 (1 mg mL^−1^) were dissolved in CA solutions with fixed concentrations of 0.25, 0.5, and 1 mg mL^−1^, respectively. Following the same protocol of TCP coatings, the thickness, particle size, PDI were recorded at preset time. The pH was adjusted by 0.1 m NaOH or 0.1 m HCl.

### Characterization

The thickness of the coating was measured by a multi‐angle ellipsometry polarization spectrometer (Woollam M2000UI, J. A. Woollam Co., Inc., Lincoln, NE) at two incidence angles of 65° and 70°, and analyzed by the WVASE32 analysis software. Fourier transform infrared (FTIR) spectroscopy of the coatings was performed by a Tensor II spectrometer (Bruker, Germany). The sample was scanned in the wavenumber range of 400–3600 cm^−1^ using attenuated total reflection mode (ATR), and the data were baseline corrected using the instrument companion software. The assembled TCP coatings were characterized by X‐ray photoelectron spectroscopy (XPS, ESCALAB 250), and peak data were obtained at a starting angle of 90° and through energy of 20 eV. The absorbance of coatings at different assembly times was studied using a UV–vis near‐infrared spectrometer (UV, CARY5000). The surface morphology of the coating was characterized by field emission scanning electron microscopy (SEM, SU8010, HITACHI). The particle size of the particles in the assembly solution was analyzed by the nanoparticle size analyzer (ZEN3600, Malvern). The water contact angle was measured using a goniometer (Bioline Sweden). The roughness of the coating surface was characterized using an Atomic Force Microscope (AFM, Multi‐Mode 8).

### Molecular Dynamics (MD) Simulations

The initial structures for the MD simulations were constructed by the insert‐molecules module of Gromacs to build a hybrid system of three molecules randomly populated into 8 × 8 × 8 nm boxes with six of each of the three molecules. The MD simulations were performed using the Gromacs 2021.5 program at constant temperature and pressure as well as under periodic boundary conditions. The Amber14SB all‐atom force field, TIP3P water model was applied. During MD simulations, all involved hydrogen bonds were constrained using the LINCS algorithm with an integration step of 2 fs. Electrostatic interactions were calculated using the (Particle‐mesh Ewald) PME method. The non‐bonded interaction cut‐off was set to 12 Å and updated every 10 steps. The V‐rescale temperature coupling method was used to control the simulation temperature to 300 K, and the Parrinello‐Rahman method was used to control the pressure to 1 bar. Firstly, the system was subjected to energy minimization using the steepest descent method in order to eliminate too close contacts between atoms; then, NVT equilibrium simulations were carried out at 300 K for 100 ps; finally, the system was subjected to 200 ns MD simulations were performed at 300 K for 100 ps; finally, 200 ns MD simulations were performed for the system, and the conformations were saved every 40 ps, with a total of 5000 conformations; the visualization of the simulation results was done by using the Gromacs embedded program and VMD.

### Intermolecular Interaction Studies

The general program in Molclus software was used to search for the cluster structures of TA‐H6‐CA ternary complexes respectively, the system was collected for 100 initial conformations and the initial structures were first processed for structure optimization using the xTB 6.3.0 program at the gfn2 level, then the optimized structures were processed for structure optimization using the ORCA 5.0.1 program at the B3LYP ‐D3/def2svp conditions for the structure optimization process, and then all conformations were analyzed in clusters, with the energy threshold set at 1 kca/mol and the geometrical structure threshold set at 2 Å, and the first six clusters with the lowest energies were taken for the subsequent calculations. For the cluster structures obtained by Molclus, the intermolecular interaction energies were calculated using the ORCA program at the B3LYP‐D3/def2‐TZVP level, with the addition of Basis Set Superposition Error (BSSE) to the calculation to be more positive. The binding energy can be calculated using the following equation: G_bind = G(AB)‐G(A)‐G(B)+BSSE, where, G(AB) was the energy of the complex, G(A) was the energy of the template molecule, G(B) was the energy of the monomer molecule, and BSSE indicates the corrected energy of the system. (Molecular simulation and intermolecular interactions were calculated by Sichuan Mode Technology Co).

### Intermolecular Interactions in TCH Coatings

The stability of TCP coatings was evaluated by the TCH coatings. The as‐prepared TCH coatings with a thickness of 400 nm were immersed in 100 mm NaCl solution, 100 mm Urea solution, 100 mm Tween solution, and buffer solution (100 mm HAc‐NaAc buffer) with pH at 3, 5, and 7, respectively. At the preset immersed time, the thickness of the TCH coating was recorded by ellipsometry. The pH was adjusted by 0.1 m NaOH or 0.1 m HCl.

### Cell Experiments

MC3T3‐E1 cells derived from mouse bone marrow were purchased from Punosi and cultured in α‐MEM basic medium supplemented with 100 U mL^−1^ penicillin, 100 U mL^−1^ streptomycin, and 10% FBS at 37 °C with 5% carbon dioxide. On the second day, cells were cultured in a 25 mm^2^ flask and grown to an 80% coverage.

Cell proliferation was tested on TCH coatings, TCH‐RGD coatings, and glass coverslips (control) by seeding MC3T3‐E1 cells at a density of 1× 10^4^ cells in 24‐well plates. After culturing for preset days (1, 3, and 7), the CCK‐8 kit was used to test the viability of cells by the protocol provided by the kit manufacturer. Live/dead staining kits were used to test the biocompatibility of TCH coatings and TCH‐RGD coatings after 2 days of seeding by the protocol provided by the kit manufacturer.

For the early adhesion and diffusion of cells, MC3T3‐E1 cells were seeded on Ti wafers (control), and Ti wafers coated with TCH and TCH‐RGD at a density of 1× 10^4^ in 24‐well plates. After t 2 and 4 h incubation, the samples were removed, gently rinsed with PBS, and then soaked in 4% paraformaldehyde for 30 min to fix the cells. The cells were dehydrated with ethanol solutions of different concentration gradients and then dried. Cellular morphology was observed by SEM.

For osteogenic differentiation assay, MC3T3‐E1 cells were seeded on glass coverslips (control), and glass coverslips coated with TCH and TCH‐RGD at a density of 1× 10^4^ in 24‐well plates. An osteogenic induction medium was used once cell confluence was over 80%. Early osteogenic differentiation was stained with alkaline phosphatase (ALP) activity by the protocol provided by the kit manufacturer. On days 7 and 14, cells were fixed with 4% paraformaldehyde and then visualized by microscopy.

### Antimicrobial Test

For the antimicrobial test, the glass slide, TCH coating, and TCH‐AMP coating were incubated into 1 mL S. aureus and E. coli with a concentration of 107 colony‐forming units (CFU) per milliliter, for 8 h at 37 °C. Then, the glass slide, TCH coating, and TCH‐AMP coating were gently washed three times with PBS. Subsequently, the glass slide, TCH coatings, and TCH‐AMP coatings were subjected to sonication in 3 mL of PBS solution for 8 min and vortexing for 15 s, thereby facilitating the release of the attached bacteria into the solution. The isolated bacterial solution was then diluted with PBS solution and applied to the TSB‐agar plate. Following a 24 h incubation period at 37 °C, the number of bacterial colonies was counted.

For the bacterial live/dead test, a lower *S. aureus* and *E. coli* concentration of 105 CFU was applied for incubating with the glass slide, TCH coating, and TCH‐AMP coating for 8 h at 37 °C. After being gently washed three times with PBS, the S. aureus and E. coli were stained by bacterial live/dead kit, with the protocol provided by the manufacturer. Then observed using an inverted fluorescence microscope, and the images were captured in randomly selected fields of view.

### Protein Adsorption Behavior

Glass coverslips, TCH‐coated and TCH‐PEG2k coated glass coverslips were immersed in 1 mL of fluorescently labeled 1 mg mL^−1^ bovine serum albumin (BSA) or 1 mg mL^−1^ lysozyme (Lys) for 2 d. Then remove the sample and rinse it twice with water, followed by taking photos under a fluorescence microscope. Anti‐protein adhesion was measured by the statistical fluorescence intensity from at least 20 images, and the average values were reported.

### Statistics Analysis

SPSS 21.0 software was used for the statistics analysis. One‐way ANOVA was chosen for comparing differences among different groups. For the post hoc tests, Student–Newman–Keuls method was used. The difference significance was treated as: *p < 0.05, **p < 0.01.

## Conflict of Interest

The authors declare no conflict of interest.

## Author Contributions

K.H., Z.S., N.L., and Q.W. investigated, designed the study, and wrote the original manuscript. K.H., Z.S., and W.L. performed most experiments. W.L. and H.L. conducted AFM‐related tests. K.H., Q.W., and Y.C. analyzed data with intellectual contributions. X.Z. and N.L. revised the manuscript. Q.W., X.Z., and N.L. supervised this work and acquired the funding. All authors have read and approved the final manuscript for submission.

## Supporting information



Supporting Information

## Data Availability

The The data that support the findings of this study are available from the corresponding author upon reasonable request.;
