# Supplementary material for: Modulating the Interactions of Peptide‐Polyphenol for Supramolecular Assembly Coatings with Controllable Kinetics and Multifunctionalities
Source: Adv Sci (Weinh). 2024 Nov 25;12(3):2412194. doi: 10.1002/advs.202412194 (PMC11744643; doi:10.1002/advs.202412194)
Supplement: Supplementary file 1 — Supporting Information [file ADVS-12-2412194-s001.pdf]

ADVANCED SCIENCE
Open Access

Supporting Information

for *Adv. Sci.*, DOI 10.1002/advs.202412194

Modulating the Interactions of Peptide-Polyphenol for Supramolecular Assembly Coatings with Controllable Kinetics and Multifunctionalities

Kaiyuan Huo, Wenjie Liu, Zeyu Shou, Hongxiang Wang, Hao Liu, Yang Chen, Xingjie Zan, Qing Wang* and Na Li**

Supplementary Information

Modulating the Interactions of Peptide-Polyphenol for Supramolecular Assembly Coatings with Controllable Kinetics and Multifunctionalities

Kaiyuan Huo,[¶,♀] *Wenjie Liu,*[♀,Ψ] *Zeyu Shou,*[♀,ϕ,§] *Hongxiang Wang,*[Ƥ] *Hao Liu,*[Ψ] *Yang Chen,*[¶] *Xingjie Zan,*[¶,♀]* *Qing Wang*[Ƭ], *Na Li,*[¶,♀,Ƥ]*

[¶] School of Ophthalmology and Optometry, School of Biomedical Engineering, Wenzhou Medical University, Wenzhou, Zhejiang 325027, China
[♀] Wenzhou Institute, University of Chinese Academy of Sciences, Wenzhou Key Laboratory of Perioperative Medicine, Wenzhou, Zhejiang, 325001, China
[Ψ] School of Materials Science and Engineering, Zhengzhou University, Zhengzhou 450001, China
[ϕ] Department of Orthopedics, The People's Hospital of Zhuji, Affiliated Zhuji Hospital, Wenzhou Medical University, Shaoxing, Zhejiang, 311800, China
[§] Department of Orthopedics, The First Affiliated Hospital of Wenzhou Medical University, Wenzhou, Zhejiang, 325000, China
[Ƭ] Yongkang First People's Hospital of Wenzhou Medical University， Yongkang, Zhejiang，321300，China
[Ƥ] School of Pharmacy, Zhejiang Chinese Medical University, Hangzhou, Zhejiang, 310053 China

*Corresponding author. E-mail: zanxj@ucas.ac.cn (X. Zan); lina0701@ucas.ac.cn (N. Li)

polyphenols	tannic acid	TA
	proanthocyanidins	PC
	epigallocatechin gallate	EGCG
	catechins	CAT
	gallic acid	GA
organic acids	citric acid	CA
	tartaric acid	TAa
	oxalic acid	OA
	acetate acid	HAc
	ethylenediaminetetraacetic acid	EDTA
polypeptide	aspartate-6	Asp6
	glycine-6	Gly6
	isoleucine-6	Ile6
	phenylalanine-6	Phe6
	proline-6	Pro6
	lysine-6	Lys6
	arginine-6	Arg6
	histidine-2	H2
	histidine-3	H3
	histidine-6	H6
	histidine-9	H9
	H6-GGYGFGGYGFGG	H6-OGP
	H6Cys-mal-PEG2k	H6-PEG2k
	H6-antimicrobial peptide	H6-AMP
	H6-Acp-RGD-Acp-RGD-Acp-RGD	H6-RGD
coating	TA-CA-H6 coating	TCH
	TA-CA-Lys6 coating	TCK
	TA-CA-Arg6 coating	TCR
	TA-CA-H6-OGP	TCH-OGP
	TA-CA-H6-PEG2k	TCH-PEG2k
	TA-CA-H6-AMP	TCH-AMP
	TA-CA-H6-RGD	TCH-RGD
	TA-CA-polypeptide	TCP

Table S1. Abbreviations of the materials used in this work and the names of the prepared coatings.

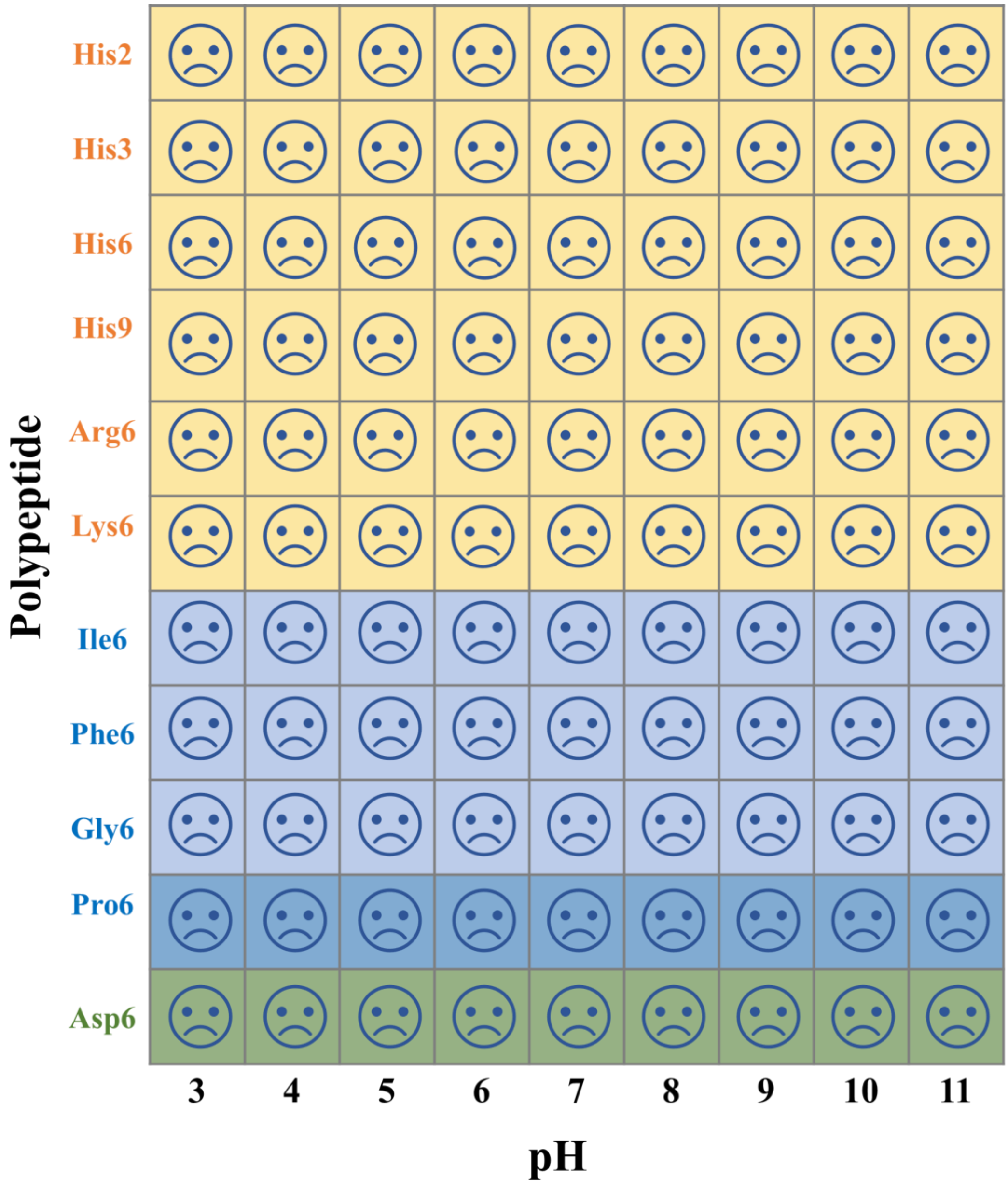

Figure S1. TA (3 mg/mL) and various peptides (3 mg/mL) were dissolved in water. Both solutions were adjusted to pH=4 with 0.1 M NaOH and 0.1 M HCl. 0.4 mL of TA

solution and 0.4 mL of peptide solution were transferred to a 1.5 mL centrifuge tube (fix the mass ratio of TA to peptide to be 1:1). Vortex for 10 seconds. The substrate was placed into the mixed solution and the growth of the coating was evaluated by ellipsometry. Smiling face indicates successful construction of the coating, crying face indicates unsuccessful construction of the coating, yellow area indicates positively charged peptide, blue indicates uncharged peptide, and green area indicates negatively charged peptide.

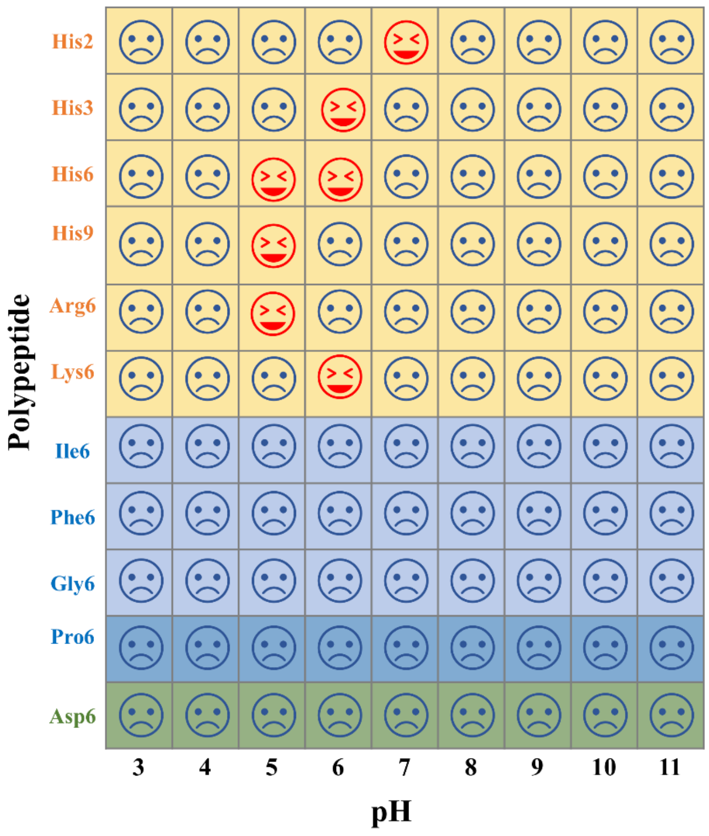

Figure S2. TA (3 mg/mL) + CA (1.5 mg/mL), various peptides (3 mg/mL) + CA (1.5 mg/mL) were dissolved in water. Both solutions were adjusted to pH=4 with 0.1 M NaOH and 0.1 M HCl. 0.4 mL of TA solution and 0.4 mL of peptide solution were transferred to a 1.5 mL centrifuge tube, (the mass ratio of TA, CA to peptide was fixed at 1:1:1). Vortex for 10 seconds. The substrate was placed into the mixed solution and the growth of the coating was evaluated by ellipsometry. Smiling face indicates that the coating was successfully constructed, crying face indicates that the coating was not successfully constructed, yellow area indicates positively charged peptides, blue indicates uncharged peptides, and green area indicates negatively charged peptides.

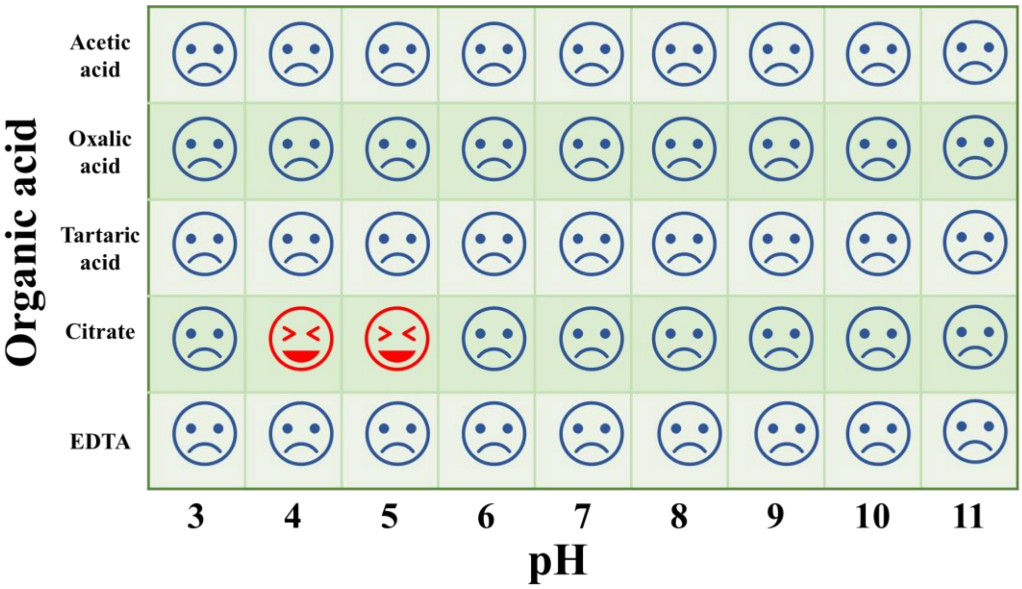

Figure S3. TA (6 mg/mL) and H6 (6 mg/mL) were dissolved in water, and then an equal volume of organic acid conditioner (3 mg/mL) was added to each solution. Both solutions were adjusted to pH=4 with 0.1 M NaOH and 0.1 M HCl. 0.4 mL of each solution was transferred to a 1.5 mL centrifuge tube (the mass ratio of TA, organic acid to H6 was fixed at 1:1:1). Vortex for 10 seconds. The substrate was placed into the mixed solution and the growth of the coating was evaluated by ellipsometry. A smiling face indicates successful construction of the coating and a crying face indicates unsuccessful construction of the coating.

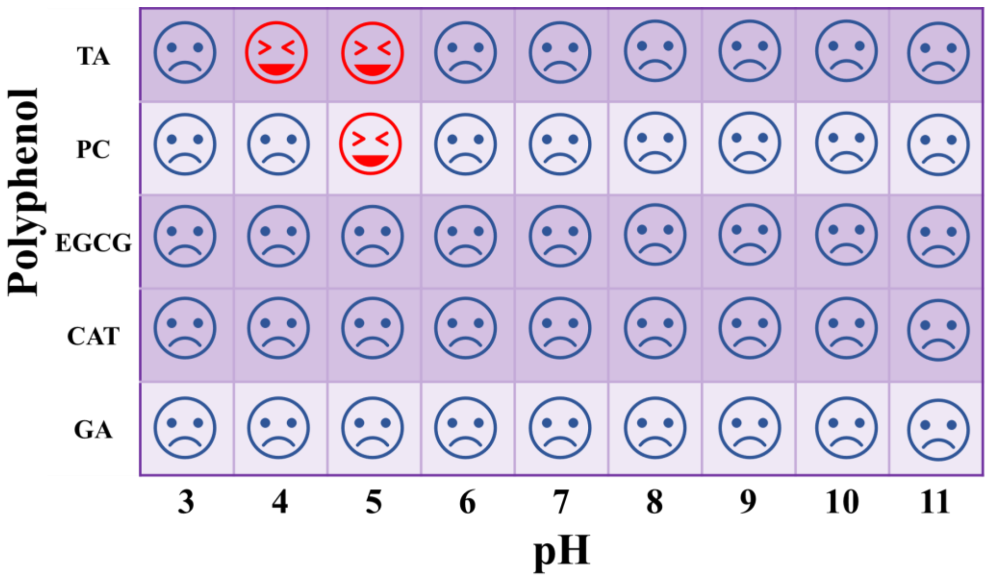

Figure S4. Various polyphenols (3 mg/mL) + CA (1.5 mg/mL) 、 H6 (3 mg/mL) + CA (1.5 mg/mL) were dissolved in water. Both solutions were adjusted to pH=4 with 0.1 M NaOH and 0.1 M HCl. 0.4 mL of polyphenol solution and 0.4 mL of H6 solution were transferred to a 1.5 mL centrifuge tube (the mass ratio of polyphenol, CA, and H6 was fixed at 1:1:1). Vortex for 10 seconds. The substrate was placed into the mixed solution and the growth of the coating was evaluated by ellipsometry. Smiling face indicates that the coating was successfully constructed, crying face indicates that the coating was not successfully constructed.

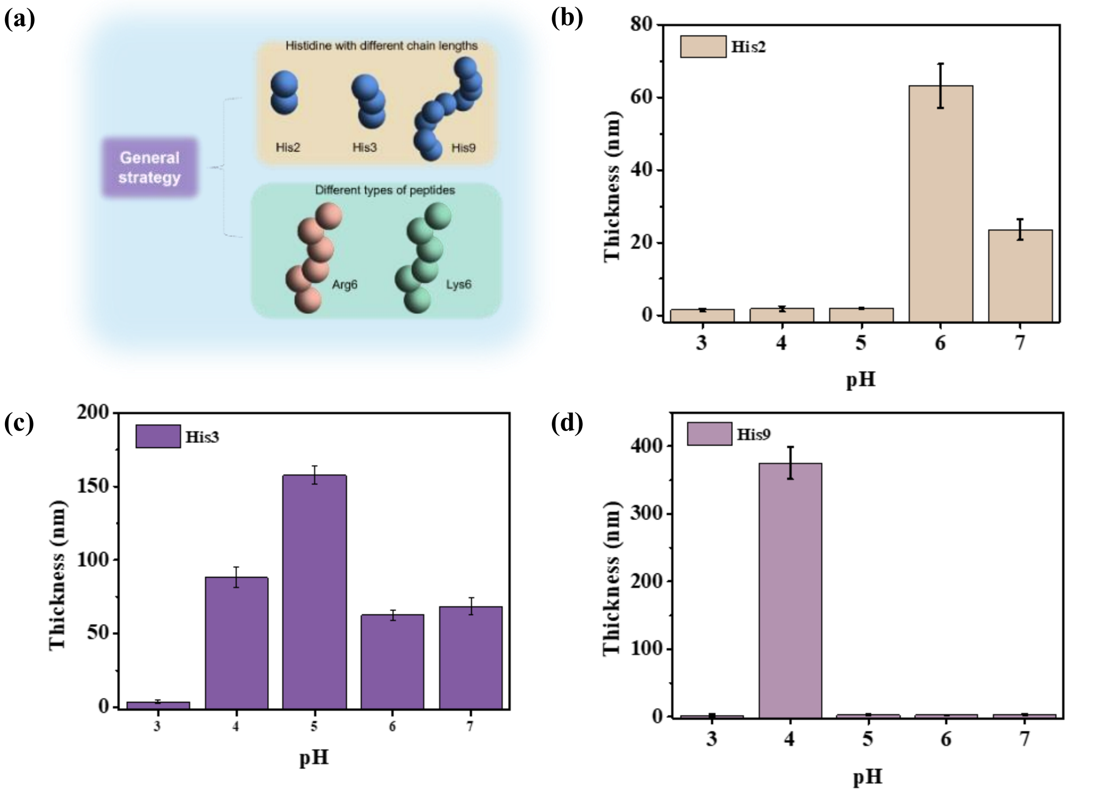

Figure S5. (a) Schematic illustration of the generality of the TCP coating strategy. (b) Assembly thickness of His2/ (c) His3/ (d) His9 under different pH conditions.

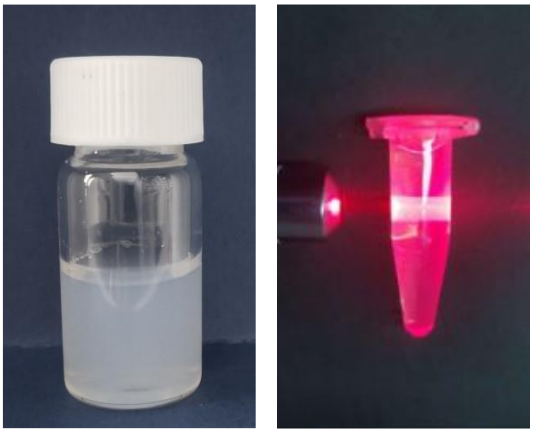

Figure S6. TCH coating in the initial stage of assembly solution (left image). The solution exhibits a distinct Tyndall effect (right image).

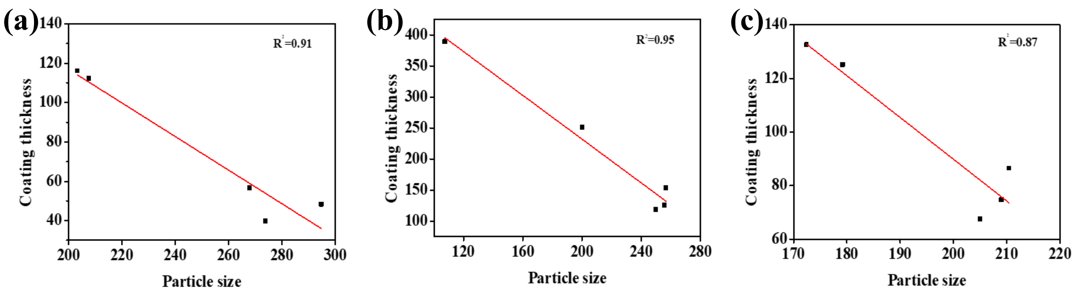

Figure S7. Correlation between particle onset size and tissue coating thickness in assembled solutions at CA concentrations of (a) 0.25 mg/mL, (b) 0.5 mg/mL, and (c) 1 mg/mL.

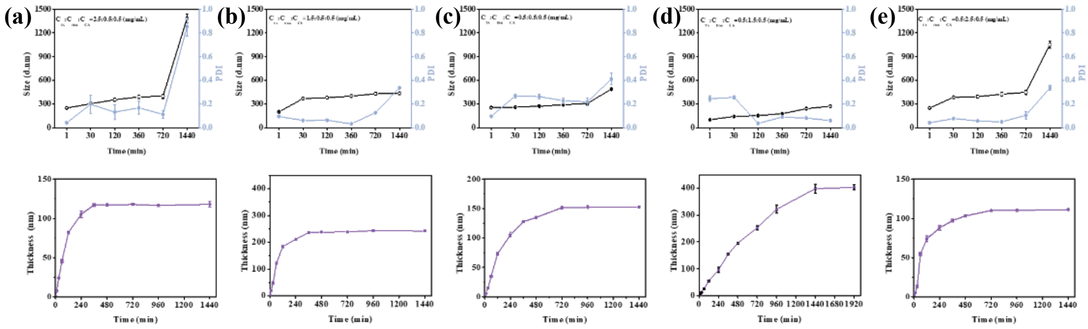

Figure S8. When the CA concentration is 0.5 mg/mL, and the concentration ratio of TA to His6 is (a) 2.5:0.5; (b) 1.5:0.5; (c) 0.5:0.5; (d) 0.5:1.5; and (e) 0.5:2.5, the curves of particle size and PDI value changes over time in the assembly solution, as well as the growth curve of the TCH coating.

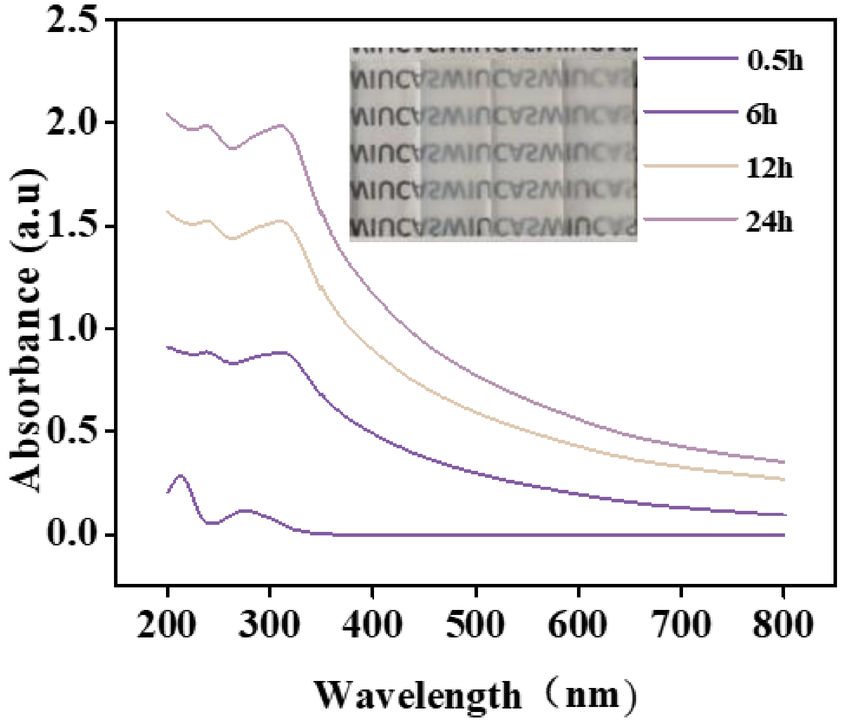

Figure S9. UV absorption curves of TCH coatings assembled for different times.

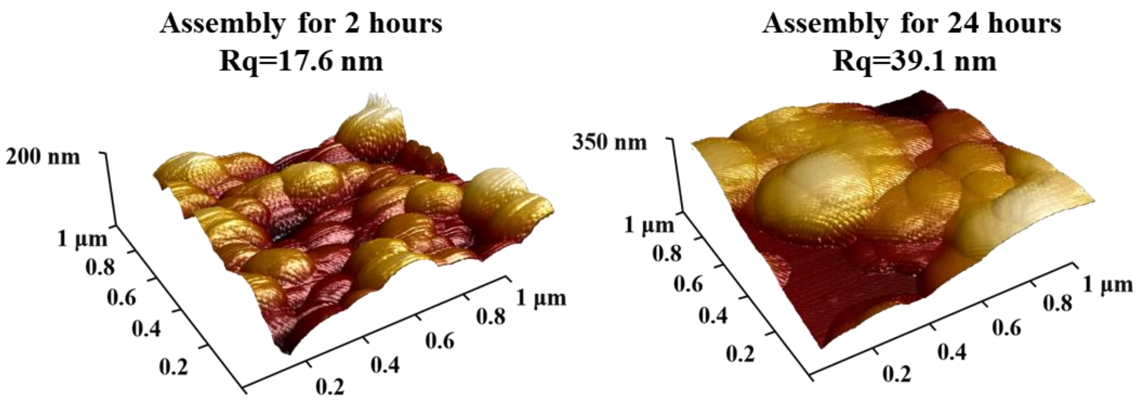

Figure S10. Image of AFM 3D height map for Assembled 2 and 24 hours of TCH coating.

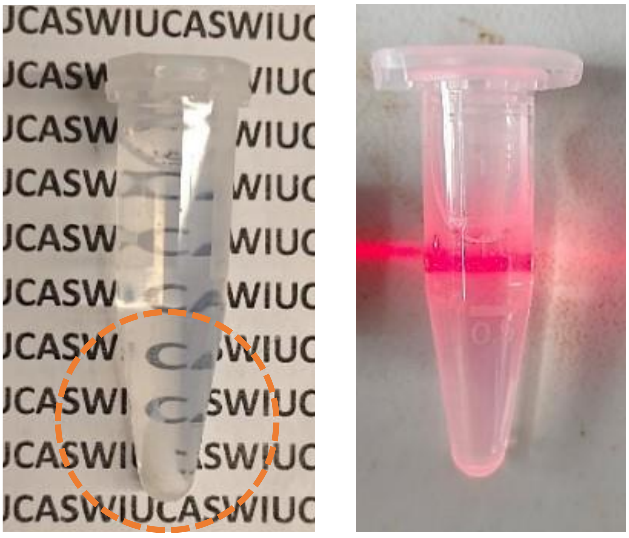

Figure S11. After centrifugation of the TCH assembly solution, a pellet was evident at the bottom of the centrifuge tube, and the supernatant was essentially clear but there was a clear Tyndall effect.

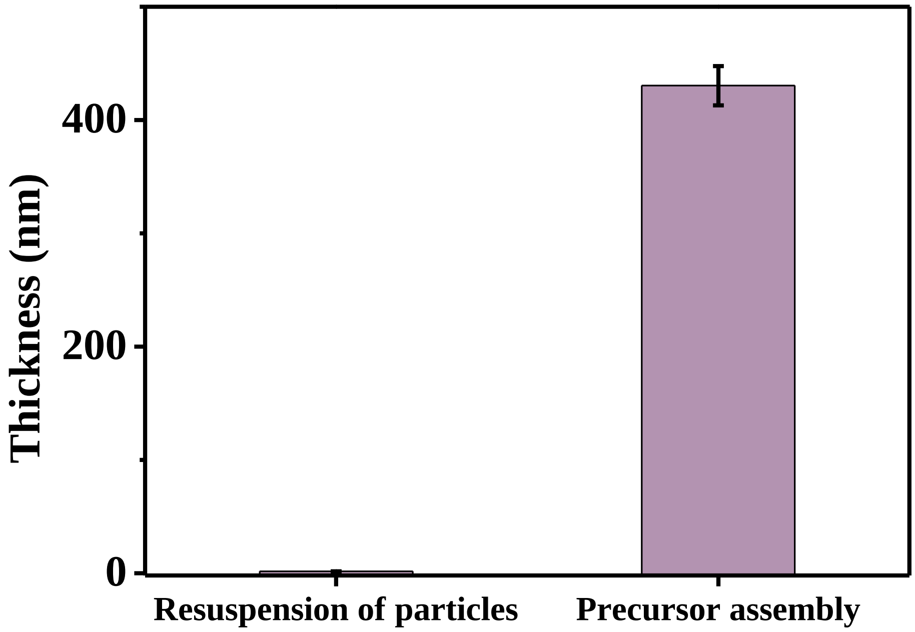

Figure S12. The TCH assembly solution was centrifuged to separate the supernatant from the pellet and resuspend the pellet. The silicon substrate was then assembled in the resuspended pellet and in the supernatant. The figure shows the final thickness of the assembly.

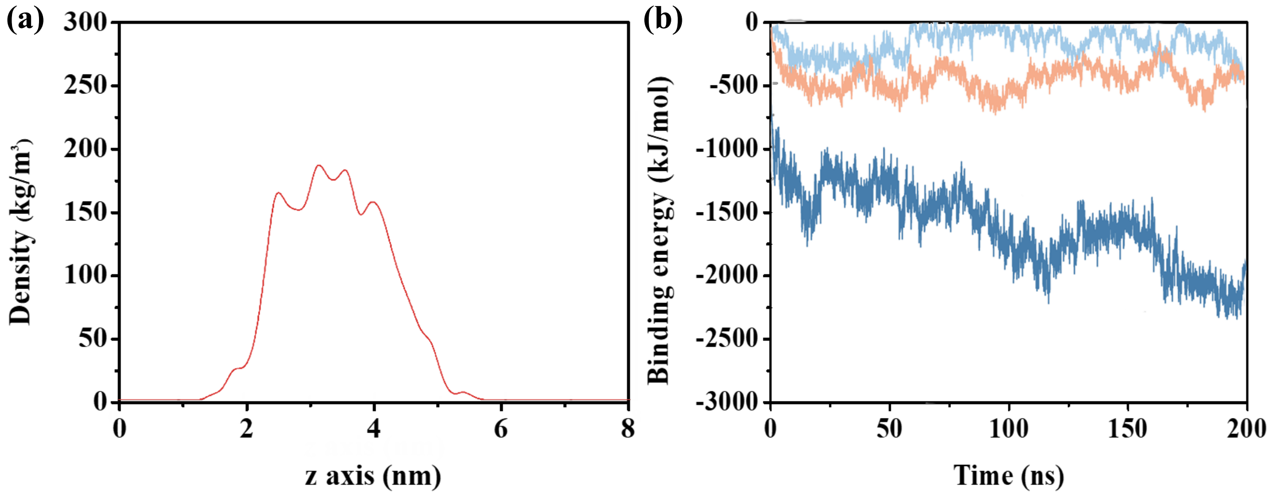

Figure S13. (a) Density distribution of assemblies in the system. (b) Intermolecular interaction energy changes during MD simulations.

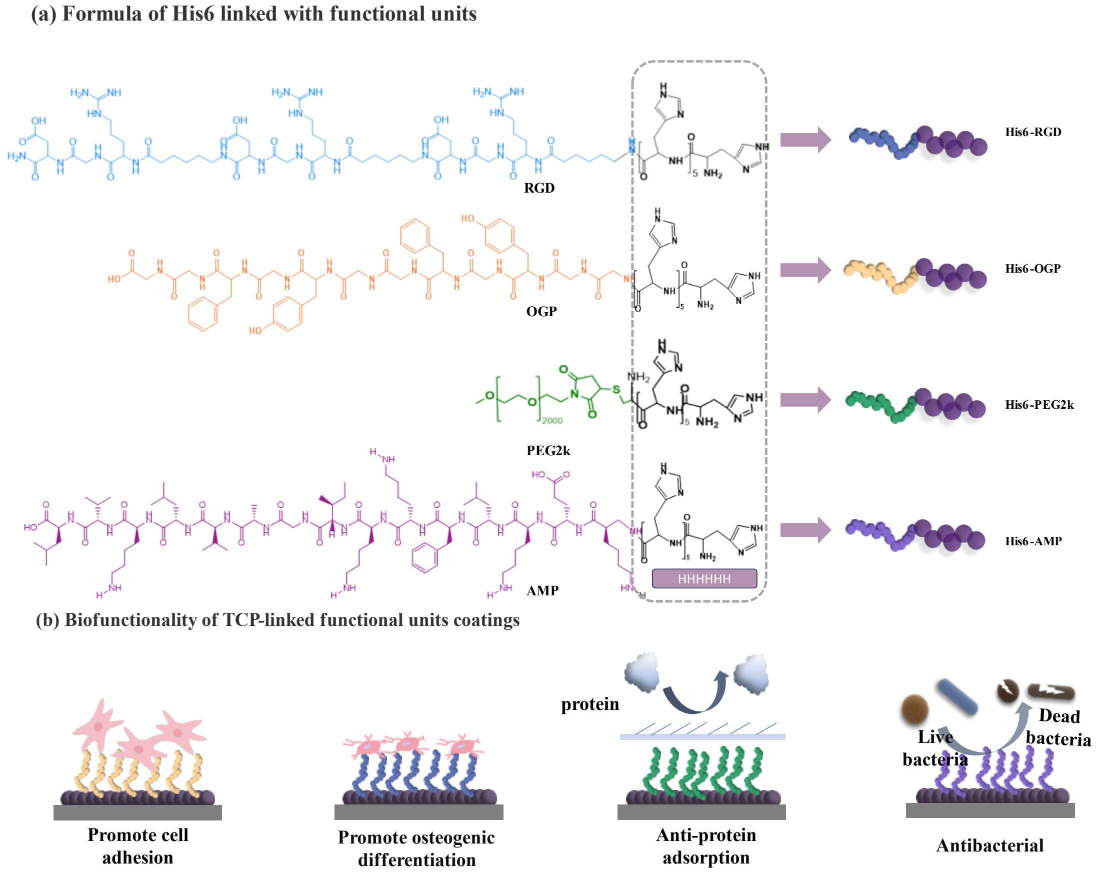

Figure S14. (a) Molecular structural formula of His6 linked with functional units. (b) Schematic diagram of the biological functions of the grafted assembly coating.

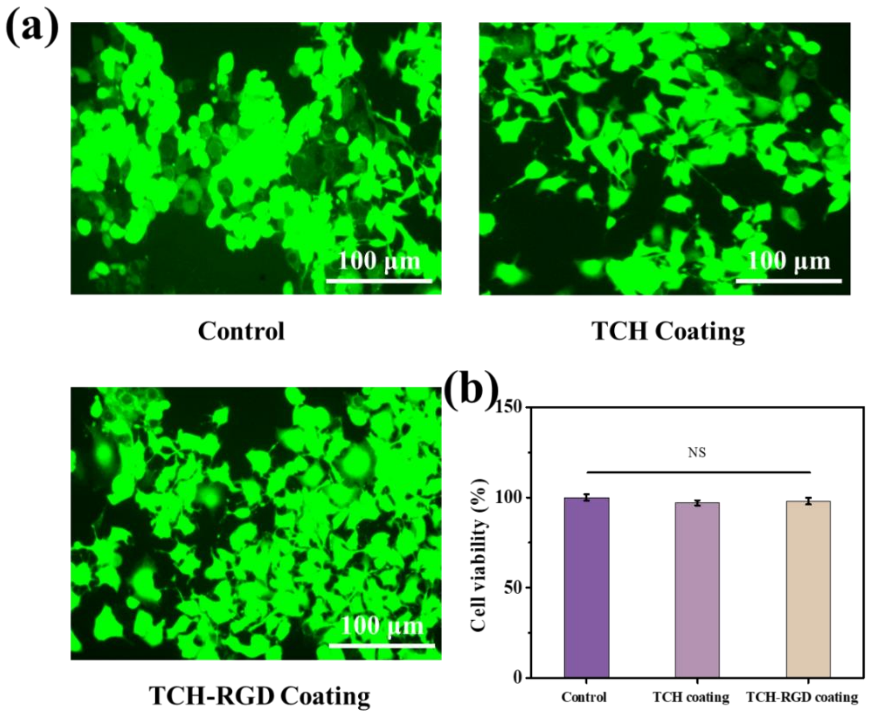

Figure S15. (a) Cell live/dead staining results and (b) statistics. No significance noted as "NS," $*p < 0.05$, $**p < 0.01$, $***p < 0.001$ compared with the Control group, using t-test.

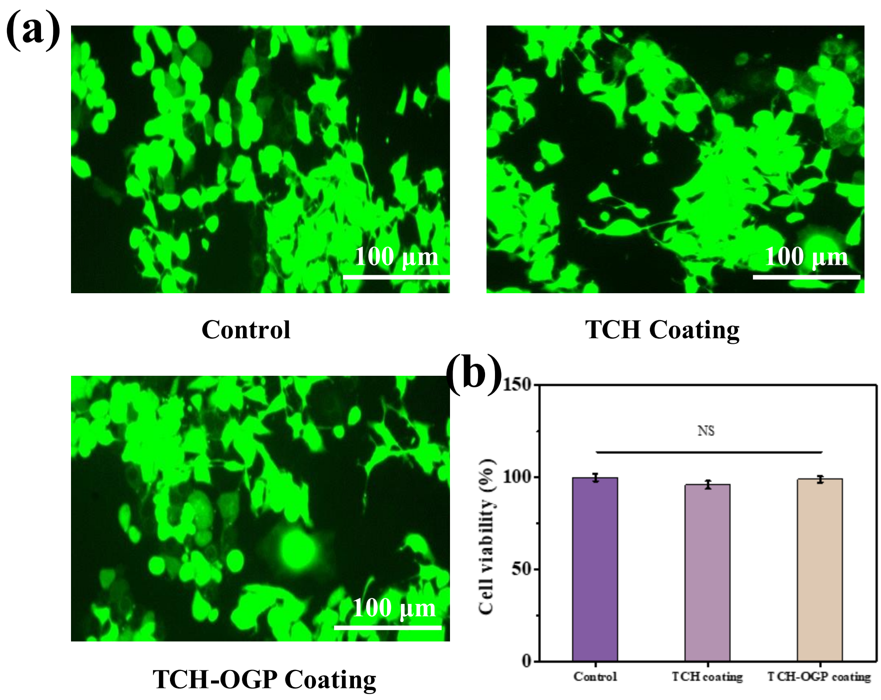

Figure S16. (a) Cell live/dead staining results and (b) statistics. No significance noted as "NS," $*p < 0.05$, $**p < 0.01$, $***p < 0.001$ compared with the Control group, using t-test.

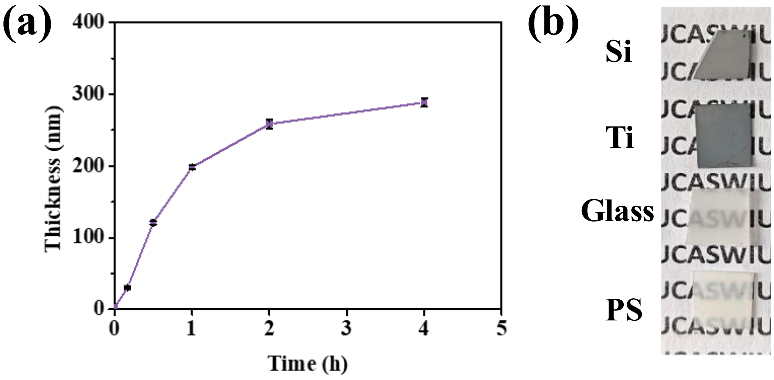

Figure S17. (a) The dependence of thickness on TCH-PEG2k coating on the incubation time, the TCH- PEG2k coating was fabricated by immersing in a mixed solution of TA (1.5mg/mL), CA (0.5mg/mL) and His6- PEG2k (0.5mg/mL), at pH 4. (b) Digital photographs of TCH-PEG2k coatings assembled on different substrates.

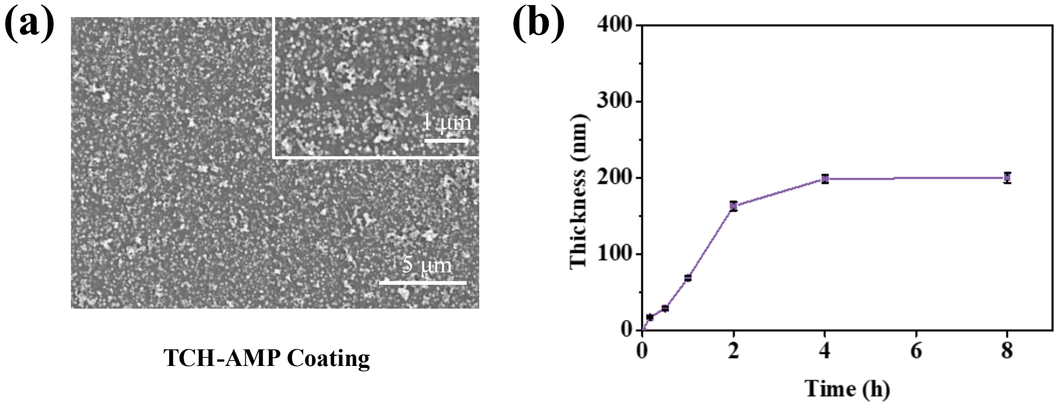

Figure S18. (a) SEM image of TCH-AMP coating. (b) Dependence of TCH-AMP coating thickness on incubation time, prepared by immersing silica in a mixed solution of TA (1.5mg/mL), CA (0.5mg/mL), and His 6-AMP (0.5mg/mL) at a pH value of 4. In Figure (a), The scale bare was 5 µm in image and 1µm for magnified image.
